# Synthesis and evaluation of pyridine-3-carboxamide analogs as effective agents against bacterial wilt in tomatoes

**DOI:** 10.1038/s41598-024-59609-z

**Published:** 2024-05-15

**Authors:** Yasser Hussein Issa Mohammed, Israa M. Shamkh, Ahmed Hassen Shntaif, Muhammad Sufyan, Md Tabish Rehman, Mohamed F. AlAjmi, Moayad Shahwan, Saad Alghamdi, Amal Ezzat Abd El-Lateef, Elshiekh B. Khidir, Amr S. Abouzied, Nasrin E. Khalifa, Weam M. A. Khojali, Bader Huwaimel, Dunia A. Al Farraj, Saeedah Musaed Almutairi

**Affiliations:** 1 Department of Biochemistry, Faculty of Applied Science, University of Hajjah, Hajjah, Yemen; 2 Department of Pharmacy, Faculty of Medicine and Medical Science, University of Al-Razi, Al-Razi, Yemen; 3 Botany and Microbiology Department, Faculty of Science, Cairo University, Giza, Egypt; 4 Chemo and Bioinformatics Lab, Bio Search Research Institution, BSRI, Giza, Egypt; 5 Department of Chemistry, College of Science for Women, University of Babylon, Alhilla, 51002 Iraq; 6 Department of Bioinformatics and Biotechnology, Government College University, Faisalabad, Pakistan; 7 Department of Pharmacognosy, College of Pharmacy, King Saud University, Riyadh, Saudi Arabia; 8 Center for Medical and Bio-Allied Health Sciences Research, Ajman University, Ajman, United Arab Emirates; 9 Department of Clinical Laboratory Sciences, Faculty of Applied Medical Sciences, Umm Al-Qura University, Makkah, Saudi Arabia; 10 Department of Pharmaceutical Chemistry, College of Pharmacy, University of Hail, 81442 Hail, Saudi Arabia; 11 Department of Pharmaceutical Chemistry, National Organization for Drug Control and Research (NODCAR), Giza, 12553 Egypt; 12 Department of Pharmaceutics, Faculty of Pharmacy, University of Khartoum, Khartoum, Sudan; 13 Department of Pharmaceutical Chemistry, Faculty of Pharmacy, Omdurman Islamic University, Omdurman, Sudan; 14 Department of Botany and Microbiology, College of Science, King Saud University, P.O. 2455, 11451 Riyadh, Saudi Arabia; 15 Clinical Pathology Department, Faculty of Medicine, Tanta University, Cairo, Egypt

**Keywords:** Docking, Pyridine‐3‐carboxamide, Bacterial wilt, Novel, Tomato, *R. solanacearum*, Biochemistry, Plant sciences

## Abstract

This study focused on developing novel pyridine-3-carboxamide analogs to treat bacterial wilt in tomatoes caused by *Ralstonia solanacearum*. The analogs were synthesized through a multistep process and their structures confirmed using spectroscopy. Molecular docking studies identified the most potent analog from the series. A specific analog, compound 4a, was found to significantly enhance disease resistance in tomato plants infected with *R. solanacearum*. The structure–activity relationship analysis showed the positions and types of substituents on the aromatic rings of compounds 4a–i strongly influenced their biological activity. Compound **4a**, with a chloro group at the para position on ring C and hydroxyl group at the ortho position on ring A, was exceptionally effective against *R. solanacearum*. When used to treat seeds, the analogs displayed remarkable efficacy, especially compound **4a** which had specific activity against bacterial wilt pathogens. Compound **4a** also promoted vegetative and reproductive growth of tomato plants, increasing seed germination and seedling vigor. In plants mechanically infected with bacteria, compound 4a substantially reduced the percentage of infection, pathogen quantity in young tissue, and disease progression. The analogs were highly potent due to their amide linkage. Molecular docking identified the best compounds with strong binding affinities. Overall, the strategic design and synthesis of these pyridine-3-carboxamide analogs offers an effective approach to targeting and controlling *R. solanacearum* and bacterial wilt in tomatoes.

## Introduction

In agricultural sector, the control on plant bacterial wilt is a challenging mission because of prevalent worldwide and effect on hundreds of plant species ^1^. Bacterial wilt incited *by R. solanacearum* infection is one of the major factor for diseases in tomato and other solanaceous plants that caused drastic loss of tomato fruit worldwide ^2–5^. The use of traditional pesticides to treat bacterial wilt in plants do not show positive effects and has been proved not very effective^6^, Therefore, discover new bioactive molecules and pesticides agent still represents a daunting task in pesticide science ^7,8^. About 450 plant species from 54 botanical families have been reported as hosts of *R. solanacearum* ^9,10^. Bacterial wilt is one of the most devastating bacterial plant diseases in tropical, sub-tropical, and warm temperate regions worldwide ^11^. Yield losses vary up to 91% in tomato, and tomato crops have high economic value; therefore, it is essential to control bacterial wilt of tomato^12^. Application of chemical plant defense activators and antagonistic microorganisms, have reduced the tomato bacterial wilt^13^. Control measures against *R. solanacearum* have long relied on traditional chemical pesticides (e.g., zinc thiazole, bismerthiazol, and saisentong) and antibiotics in many countries^14^. Chemical pesticide is a common method to control *R. solanacearum* to date. However, the negative impact of using chemical pesticides in terms of environment, health and economy have been reported everywhere in the world^15^. Streptomycin is widely used in agriculture, but the overuse of it can lead to bacterial resistance^16^. Thus, it is very necessary to search for more potent anti-*R. solanacearum* compounds. In this endeavor, our efforts towards the design of new anti-microbial agents are considered to be worthwhile to pursue further modifications on another moiety by appending nicotinic acid were tested against bacterial wilt pathogen of tomato^17^. Moreover, Molecular docking performed to predict the orientation of one macromolecule of protein with the synthesized compounds and then, we selected the lead when bound to each other, to form a stable complex at the atomic level^18^.

Pyridine‐3‐carboxamide is one of the most prevalent heterocyclic compounds in nature. For example, it is present in the coenzyme vitamin B6 family^19^ and in numerous alkaloids, further it plays a central role as a versatile building block in the synthesis of natural products as well as biologically active compounds. Further, pyridine bases are widely used in pharmaceutics as nicotinamides and nicotinic acid derivatives. Pyridine-3-carboxylic acid analogs are reported to show a variety of biological activities such as antiviral^20^, antibacterial^21^, antifungal^22^, pesticidal, herbicidal^23^, antiprotozoal, nematocidal, antitubercular, anticancer, local anesthetic, anticonvulsant, antioxidant, anti-inflammatory and possible antimycotic properties^24^. In this communication, we report the synthesis of newly designed *N*-(4-phenylthiazol-2-yl) nicotinamide analogs 4a–i and evaluated these compounds to provokes resistance in tomato against bacterial wilt pathogen, *R. solanacearum*.

## Results

### Interaction analysis

In this study, we report the synthesis of *N*-(4-phenylthiazol-2-yl) nicotinamide derivatives **4a–i** by conjugating 2-amino-4-phenyl thiazoles with nicotinic acids, using the concept of pharmacophore hybridization to obtain compounds that comprise both thiazole and pyridine moieties. Plant pathogens use carbohydrate–protein interactions in their strategy for host recognition, attachment, and invasion. The bacterium *Ralstonia solanacearum,* which is distributed worldwide and causes lethal wilt in many agricultural crops, was shown to produce a potent l-fucose-binding lectin, Lectins are glycan-binding proteins that are involved in the recognition of glycoconjugates at the cell surface. These Lectins were shown to have an essential role during pathogenesis in the host recognition and adhesion. To investigate the binding types between the designed compounds and the active site of the Monomeric Ralstonia solanacearum lectin (PDB: 4CSD), we conducted molecular docking studies. These studies revealed that the compound was highly effective, which led us to initiate the synthesis process. Finally, we evaluated the pharmacological activities of the synthesized compounds (**4a–i**) against the bacterial wilt pathogen *R. solanacearum*. The use of a rigorous experimental design and careful analysis allowed for the identification of promising compounds that can contribute to the development of effective disease management strategies in agriculture.

### Integrating structure–activity relationship (SAR) and molecular docking studies to Decipher the interactions of *N*-(4-phenylthiazol-2-yl) nicotinamide derivatives: implications for activity optimization and drug discovery

The efficacy of *N*-(4-phenylthiazol-2-yl) nicotinamide derivatives **4a–i** in protecting against bacterial diseases was investigated. The Structure–Activity Relationship (SAR) revealed that the bacterial wilt pathogen depended on the effect of different substituents structure of compound **4a**, that appeared highly effective in inhibiting the bacterial wilt pathogen. the electron-donating substitution linked to the C ring presented in the analogues, increase the antibacterial activity the bacterial wilt pathogen than other compounds in the series.

**Figure 1:**
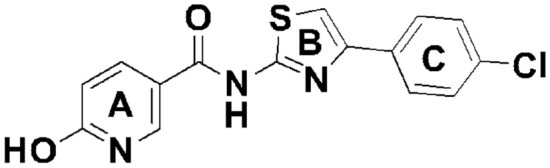
The basic structure of the compound *N*-(4-(4-chlorophenyl)thiazol-2-yl)-6-hydroxynicotinamide.

**Figure 2:**
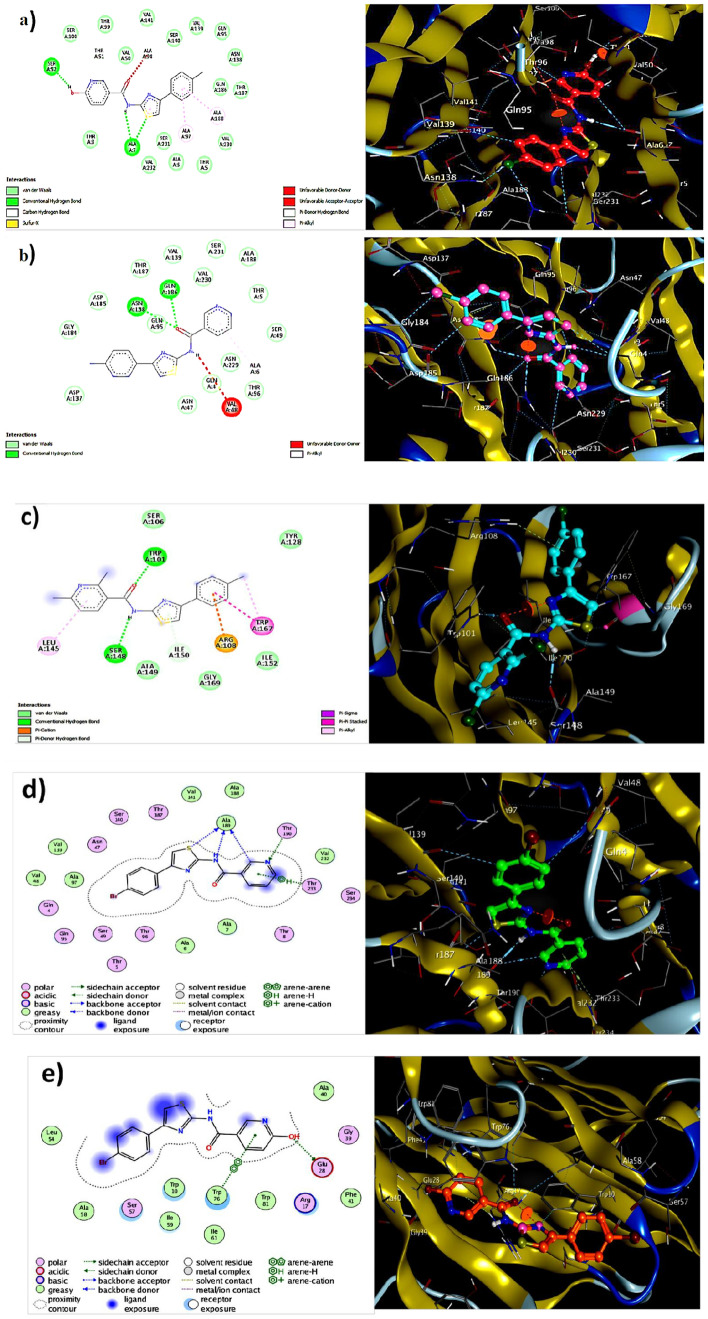

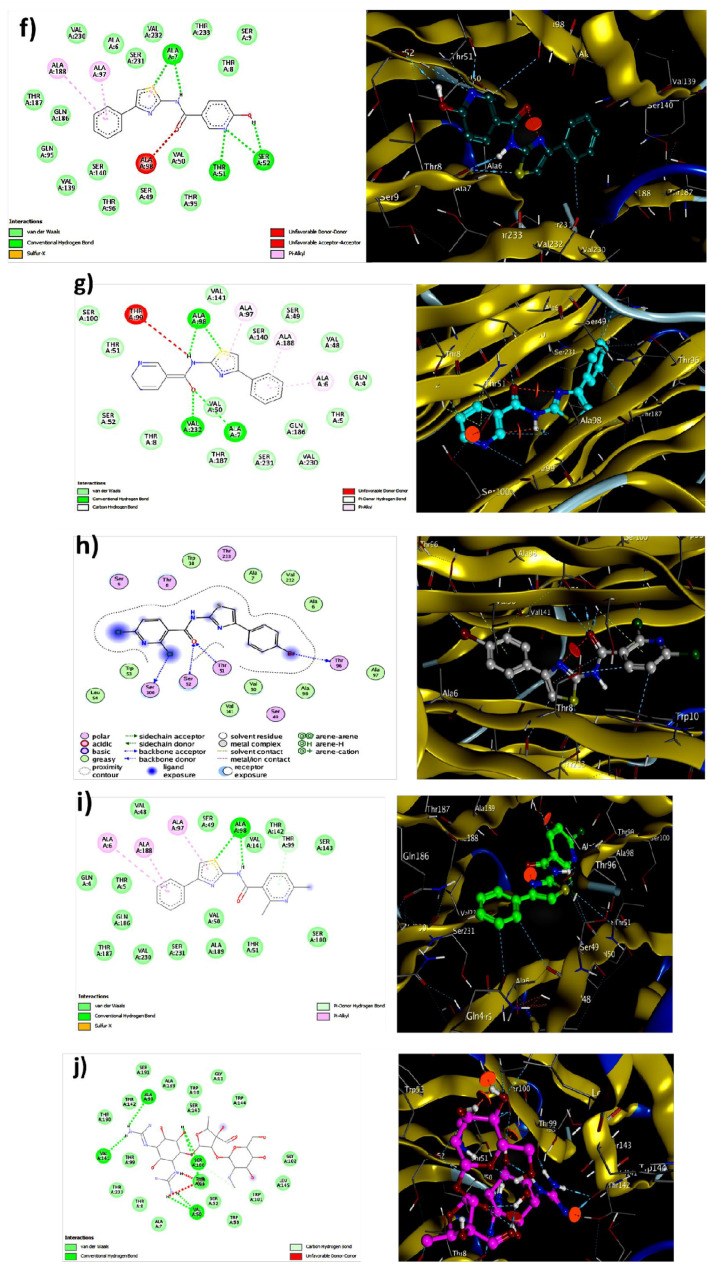
The 2D and 3D interactions poses of compounds 4a–i with 4CSD.

In the present investigation, the structure–activity relationship (SAR) mode of and inhibition extent of compounds **4a–i** against bacterial wilt pathogen insilco of *R. solanacearum* model (PDB ID: 4CSD). Compound 4a, which has a chloro-group at the para position in ring C and a hydroxy-group at the ortho position in A ring adjacent both sides of the nicotinamide ring (Fig. 1), exhibited good inhibition activity to the standard drug streptomycin. The mode of interaction was analyzed by docking using the selected active site of amino acids of the chain for *R. solanacearum* model. The compounds **4a–i** exhibited various modes of interaction with the active site of amino acids. Compound **4a** demonstrated relatively better activity against bacterial wilt pathogen (Table S1), which was supported by docking studies (Fig. 2). Overall, this study highlights the potential of *N*-(4-phenylthiazol-2-yl) nicotinamide derivatives **4a–i** as promising inhibitors of bacterial wilt pathogen and provides insights into their molecular interactions with the pathogen, which can contribute to the development of effective disease management strategies in agriculture.

The Table S1 provides information on the binding energy, ligand efficiency, inhibition constant, van der Waals (vdW) and hydrogen-bonding interaction energy, number of hydrogen bonds, amino acid residues, bonding residues, and bond length of 9 different compounds (**4a–i**) docked near the Streptomycin molecule. The compounds are being tested for their ability to bind to the same amino acid residues in the protein domain as Streptomycin, a clinically important antibiotic. The lower the binding energy of the compound, the stronger its binding to the protein, and the more effective it is in inhibiting bacterial growth. From the table, the compounds that are closest to Streptomycin in terms of their binding energy and ligand efficiency are **4a, 4d, 4e**, and **4f**. Compound **4a** has binding energy (− 8.910 kJ mol^−1^) and forms 4 H-bonds with amino acid residues such as Cys75 and Ser88 (Table S1) Therefore, compound **4a** have a higher binding energy and have hydrogen bonding interactions with the protein similar to Streptomycin, indicating that they may be effective in inhibiting bacterial growth.

### The evaluation of antimicrobial properties of synthesised compounds 4a–i against bacterial wilt pathogen

This study shows that two active compounds have inhibitory effects on the bacterial wilt pathogen. The inhibitory activity of compounds **4a–i** was determined by seed treatment of tomato seeds with different concentrations of the compounds. The effect of the seed treatment on seed quality parameters such as germination and vigor index was also evaluated and compared with a control group. the control group had a germination rate of 88 ± 0.56% and a vigor index of 1186.10 ± 3.78. The germination rate and vigor index of the treated seeds increased with increasing concentration of the compounds, indicating that the compounds have a positive effect on the seed quality parameters. the compound 4a with the highest inhibitory activity against the bacterial wilt pathogen, which at concentration of 0.001%, resulted in a germination rate of 92 ± 0.51 and a vigor index of 1613.06 ± 5.95. Moreover, the compound 4e also showed good inhibitory activity at a concentration of 0.001% (Table 1).

**Table 1:** Effect of seed treatment of **(4a–4i**) on tomato seed quality parameters values are the means + SE of three replicates.

Compounds	Concentration (%)	Germination (%)	Vigor index
Control	–	88 ± 0.56^b^	1186.10 ± 3.78^a^
	1.0	90 ± 0.57^c^	1427.66 ± 3.34^c^
4a	0.1	90 ± 0.52^c^	1530.3 ± 4.72^d^
	0.01	91 ± 0.29^de^	1586.79 ± 5.21^de^
	0.001	92 ± 0.51^de^	1613.06 ± 5.95^ef^
	1.0	89 ± 0.51^bc^	1411.86 ± 3.89^c^
4b	0.1	89 ± 1.64^bc^	1456.03 ± 4.57^bc^
	0.01	90 ± 0.51^c^	1534.63 ± 3.98^d^
	0.001	91 ± 0.78^cd^	1574 ± 4.74^d^
	1.0	88 ± 0.29^b^	1358.36 ± 3.76^b^
4c	0.1	89 ± 0.28^bc^	1433.26 ± 3.93^c^
	0.01	90 ± 0.66^c^	1537.22 ± 2.62 ^b^
	0.001	90 ± 0.57^c^	1567.61 ± 1.57 ^b^
	1.0	89 ± 0.51^bc^	1386.62 ± 4.89^b^
4d	0.1	89 ± 0.52^bc^	1446.71 ± 4.76^c^
	0.01	89 ± 1.32^bc^	1506.73 ± 3.66^d^
	0.001	90 ± 0.51^c^	1604.73 ± 4.28^ef^
	1.0	88 ± 0.78^b^	1325.8 ± 3.38^b^
4e	0.1	89 ± 0.51^bc^	1411.93 ± 4.64^bc^
	0.01	90 ± 0.51^c^	1471.5 ± 3.63^c^
	0.001	90 ± 0.57^c^	1551.93 ± 3.54^d^
	1.0	88 ± 0.51^b^	1319.86 ± 3.79^b^
4f	0.1	88 ± 0.51^b^	1367.06 ± 5.16^b^
	0.01	90 ± 1.03^c^	1500.18 ± 5.66^d^
	0.001	90 ± 0.65^c^	1536.36 ± 4.68^d^
	1.0	88 ± 0.51^b^	1347.213 ± 2.12^b^
4g	0.1	89 ± 0.53^b^	1390.38 ± 3.73^c^
	0.01	89 ± 0.51^bc^	1407.86 ± 2.63^c^
	0.001	89 ± 0.51^bc^	1495.4 ± 2.89^c^
	1.0	88 ± 1.55^b^	1413.93 ± 2.56^c^
4h	0.1	89 ± 1.03^bc^	1399.2 ± 5.26^b^
	0.01	90 ± 0.29^c^	1435.13 ± 4.96^c^
	0.001	90 ± 0.55^c^	1574.4 ± 3.18^d^
4i	1.0	87 ± 0.51^a^	1359.76 ± 4.47^c^
	0.1	88 ± 0.51^b^	1407.73 ± 4.32^c^

Duncan’s multiple range tests (DMRT): means sharing different alphabetical (a–f) superscripts in a column significantly different (P < 0.05).

The control group, which did not receive any treatment, had a germination percentage of 88 ± 0.56%, and a vigor index of 1186.10 ± 3.78. The germination percentage increased significantly in all treatments, with the highest germination percentage observed in treatment **4a** (0.001%) with 92 ± 0.51% germination. The lowest germination percentage was observed in treatment **4i** (1.0%) with 87 ± 0.51% germination. The vigor index also increased significantly in all treatments compared to the control. The highest vigor index was observed in treatment **4a** (0.001%) with a value of 1613.06 ± 5.95, while the lowest vigor index was observed in treatment **4i** (1.0%) with a value of 1359.76 ± 4.47. Overall, the results suggest that the seed treatment with compound **4a** can significantly improve the germination and vigor index of tomato seeds, followed by 4b–h, and 4i with some treatments showing better results than others.

### Validating the strong binding affinity of compound 4a with monomeric *Ralstonia solanacearum* lectin (PDB ID: 4CSD): a comprehensive study of experimental findings, molecular interactions, and implications for rational drug design

**Figure 3:**
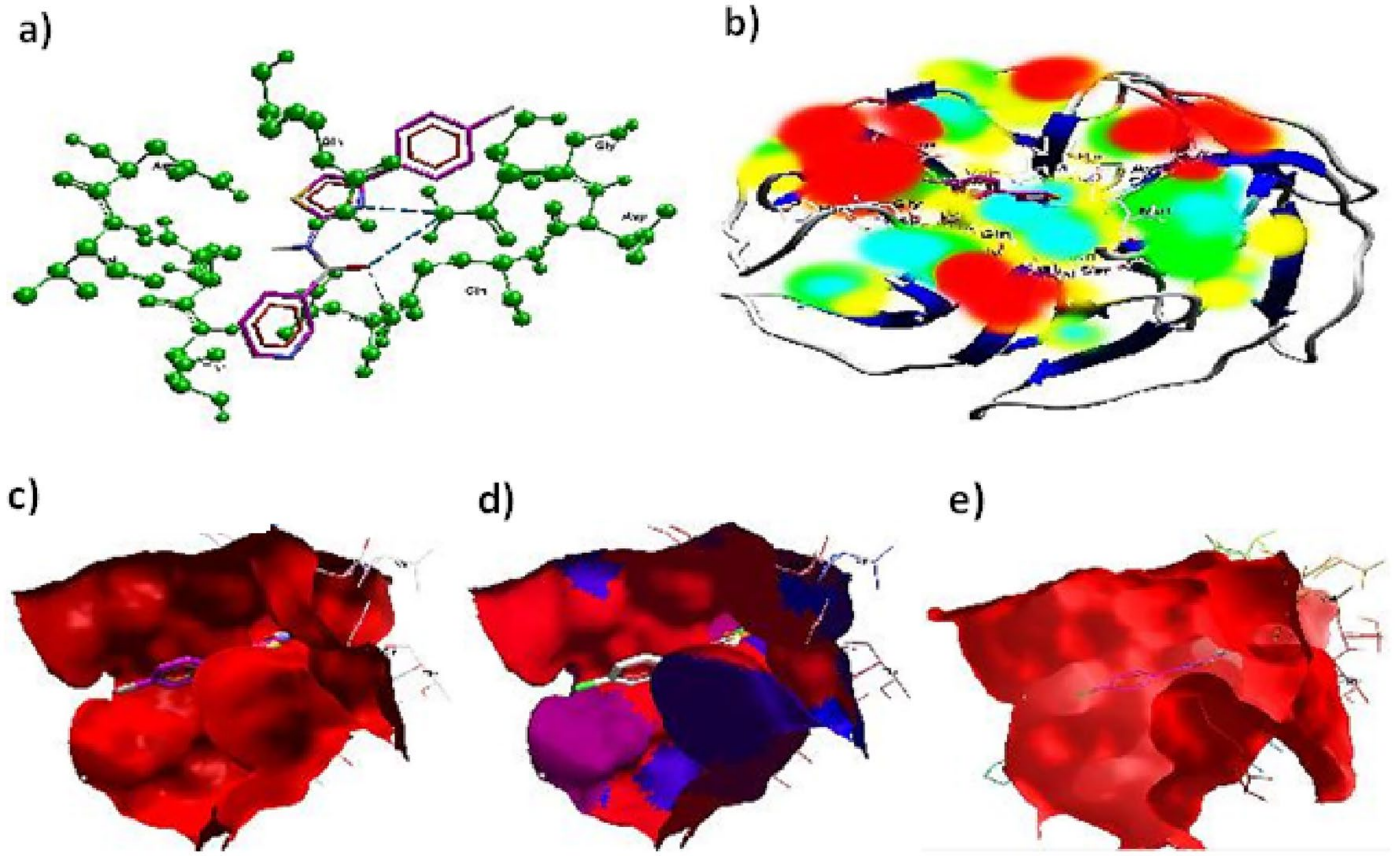
(**a**) Utilizing molecular visualization software, exhibit the organizer and viewer of compound 4a in its complex form with the amino acid residue, and delineate the hydrogen bonds formed between them. (**b**) Generate a comprehensive depiction of the molecular electrostatic potentials of compound 4a using the 4CSD database, as well as visualizing separated conformers and molecular surfaces via ribbon models. Additionally, utilize the conformer explorer to portray the interaction of compound 4a with the amino acid residue found in the 4CSD database. (**c**) Present a graphical representation of the electrostatic interactions between the 4CSD protein and compound 4a, utilizing appropriate software. (**d**) Highlight the hydrophobic and hydrophilic groups of compound 4a and the 4CSD protein by utilizing a color scheme where green is representative of hydrophobic regions and pink represents hydrophilic regions. (**e**) Adjust the preparation view to specifically emphasize and showcase the hydrogen bonds that are formed between compound **4a** and the amino acid residue.

**Figure 4:**
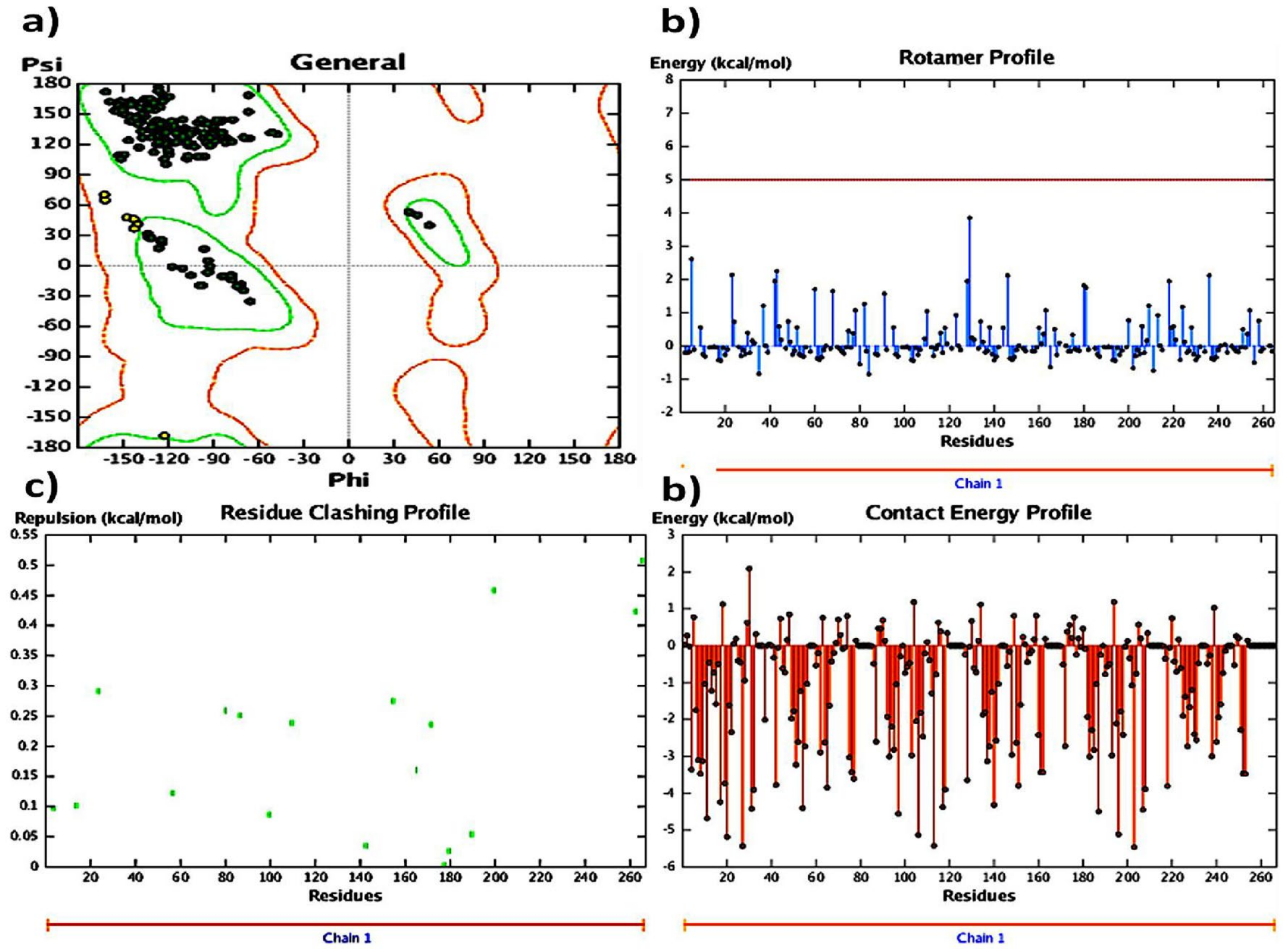
(**a**) Ramachandran plot displaying the phi/psi values of the modeled 4CSD protein. Green regions are the most favorable, red regions are allowed, pale yellow regions are generously allowed, and white regions are disallowed. (**b**) Graphical representation of the rotamer energy plots of the 4CSD protein. (**c**) Residue clashing profiles of amino acid residues between this system and others in the production stage. (**d**) The energy score (kcal mol^−1^) of the complex 4CSD-4a with the lowest binding energy of − 8.9 kcal mol^−1^.

In accordance with the established procedure for molecular docking, it was found that compound **4a**, among all compounds **4a–i**, demonstrated strong interaction with the 4CSD protein with a binding energy of − 8.910 kJ mol^−1^, as opposed to the standard binding energy of − 6.466 kJ mol^−1^ (Table S1). This finding was confirmed by analyzing the binding of compound **4a** in different conformations (Table 2). Furthermore, the 3D structure of the complex revealed the C-terminal transactivation of the interaction between compound 4a and the active site of 4CSD. Figure 3a illustrates the manner in which the ligand molecule 4a forms two hydrogen bonds with ALA 98, SER 52, and ALA 7 in the 4CSD protein. Additionally, using ribbon models and the conformer explorer, the mapping of molecular electrostatic potentials for compound 4a with the amino acid residue of the 4CSD protein was predicted (Fig. 3b). The molecular map represents a 3D plot of the electrostatic potentials that are linked to the electron density on the isoelectronic surface. It is useful to comprehend the electrophilic and nucleophilic sites, as well as their size, shape, and electrostatic potential value, which are intended for the hydrogen bond (Fig. 3c). Moreover, the interaction of compound 4a at the pocket site and residue amino acid of the complex 4CSD-4a is predicted, as are the 2D interactions of compound 4a with hydrophobic amino acids represented by green and red in the 4CSD protein (Fig. 3d). Similarly, a pose organizer viewer of the compound 4a with residue amino acids in 4CSD was performed, revealing electrostatic interactions of the 4CSD protein with the compound 4a with hydrogen view. The compound 4a displays hydrophobic and hydrophilic groups in 4CSD proteins as green and red, respectively (Fig. 3e). An illustrative depiction of the phi/psi values of the modeled 4CSD protein is presented through a Ramachandran plot. This plot exhibits the relative energy of each residue’s backbone conformational angles, phi and psi, which allows for the identification of favorable, allowed, generously allowed, and disallowed regions. The preferred regions are highlighted in green, while the permitted ones are in red, and the mildly allowed regions are in pale yellow. The disallowed regions are shown in white, indicating the regions where the protein’s backbone conformational angles are energetically unfavorable. Moreover, a graphical representation of the rotamer energy plots of the 4CSD protein is also presented. This graph illustrates the energetically favorable and unfavorable regions of the protein. It further provides a comprehensive understanding of the protein's energetics and its stability. Additionally, residue clashing profiles of amino acid residues between this system and others in the production stage are presented, showcasing the potential conflicts between the residues (Fig. 4). This profile aids in the identification of potential errors in the protein structure and helps in refining the model. Furthermore, the energy score (kcal mol^−1^) of the complex 4CSD-**4a**, which exhibits the lowest binding energy of − 8.910 kcal mol^−1^, is displayed. The lower the binding energy, the higher the stability of the protein complex. This score indicates the strength of the interaction between the protein and ligand and is an essential factor in drug discovery and design. The docking studies suggest that compound **4a** should be further investigated. Additionally, the possible binding conformations and orientations were analyzed using MOE 2015’s clustering methods. In the initial conformation of the 4CSD-4a complex, it was observed that **4a** formed hydrogen bonds with key amino acid residues in the protein. These results suggest that compound **4a** has potential as a therapeutically useful compound targeting protein catalytic sites, exhibiting favorable binding energies of − 8.910 kJ mol^−1^ (Table 2).

**Table 2:** The dock score results of the compound **4a** with 4CSD target. (PDB code: 4CSD).

Mol	Rseq	Mseq	S	Emsd_ refine	E_conf	E_place	E_ score 1	E_ refine	E_ score 2
Conformation 1	1	1	− 8.8840	2.4684	− 19.3033	− 60.2839	− 9.4980	− 40.9316	− 6.8840
Conformation 2	1	1	− 6.8026	0.9709	− 21.3683	− 74.7913	− 9.5427	− 38.5754	− 6.8026
Conformation 3	1	1	− 6.7476	0.9575	− 20.9718	− 72.7202	− 9.6802	− 36.6228	− 6.7476
Conformation 4	1	1	− 6.6964	1.3141	− 21.7927	− 59.3916	− 9.4864	− 38.6479	− 6.6964
Conformation 5	1	1	− 6.6727	1.4104	− 20.7137	− 89.4034	− 9.5191	− 34.8171	− 6.6727

### Molecular interaction of compound 4a with DNA groove: robust and favorable binding affinity and implications for drug development

**Figure 5:**
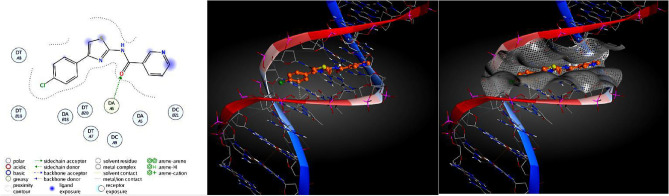
Docking simulations of DNA with compound 4a demonstrate the sphere model of the compound 4a-DNA complex positioned in the minor groove.

Our team has delved into the realm of molecular docking to explore the interaction between compound 4a and a DNA duplex of sequence d(CGCGAATTCGCG)2 dodecamer (PDB ID: 1BNA). The aim was to predict the binding site and preferred orientation of the compound within the DNA groove. Our findings, as illustrated in Table 3 and Fig. 5, reveal that the compound has a strong and attractive interaction with the narrow minor groove region of the DNA groove, mainly through its A ring. This ring, positioned within the slim A-T regions, is made possible due to the molecule’s planarity from two aromatic rings, A and C. The A moiety, with its preferential binding to A-T regions, results in van der Waals and hydrophobic interactions with DNA functional groups, thereby stabilizing the groove and the complex. The complex exhibits a “groove fit” behavior and is arranged perpendicular to the minor groove walls of the helix, stabilized by hydrogen bonding between the carbonyl group and hydroxyl groups of the A ring. Our docking simulations demonstrate that the bound compound 4a has a binding energy of − 7.2033 kJ mol^−1^ (Table 3), signifying a strong and stable interaction between the DNA receptor and **4a**, as portrayed in Fig. 5. In summary, the interaction between compound **4a** and the DNA duplex is highly favorable and predominantly involves the narrow minor groove region, stabilized by van der Waals and hydrophobic interactions, as well as hydrogen bonding. Our findings provide novel insights into the nature of compound **4a**’s interaction with DNA and its potential therapeutic applications.

**Table 3:** The dock score results of compound **4a** with a DNA duplex of the sequence d(CGCGAATTCGCG)2 dodecamer (PDB ID: 1BNA).

Mol	Rseq	mseq	S	Rmsd_refine	E_conf	E_place	E_score	E_refine	e_scor
Conformation 1	1	1	− 7.2033	0.6954	− 17.0998	− 69.8336	− 10.0852	− 41.2417	− 7.2033
Conformation 2	1	1	− 7.1447	0.8048	− 17.3679	− 69.5805	− 10.1604	− 42.3046	− 7.1447
Conformation 3	1	1	− 7.0228	0.6188	− 18.4795	− 77.2036	− 10.6224	− 41.3965	− 7.0228
Conformation 4	1	1	− 6.8723	0.9779	− 18.5500	− 66.3596	− 9.9986	− 41.0053	− 6.8723
Conformation 5	1	1	− 6.8575	0.8947	− 16.3880	− 68.7272	− 10.7846	− 37.7146	− 6.8575

### The quantum chemical properties of compound 4a: an integrated study of HOMO and LUMO energies, molecular orbitals, and implications for electronic structure analysis and rational drug design

The main utilize interpret of the biological activity and molecular properties using to improved geometry results in compound energies, this is may be attributed to the lowest unoccupied molecular orbital (LUMO) and highest occupied molecular orbital (HOMO). As from literature survey the electron donors directly depends on the HOMO and its orbital play role, while the LUMO and its orbital play role of electron acceptors.

**Figure 6:**
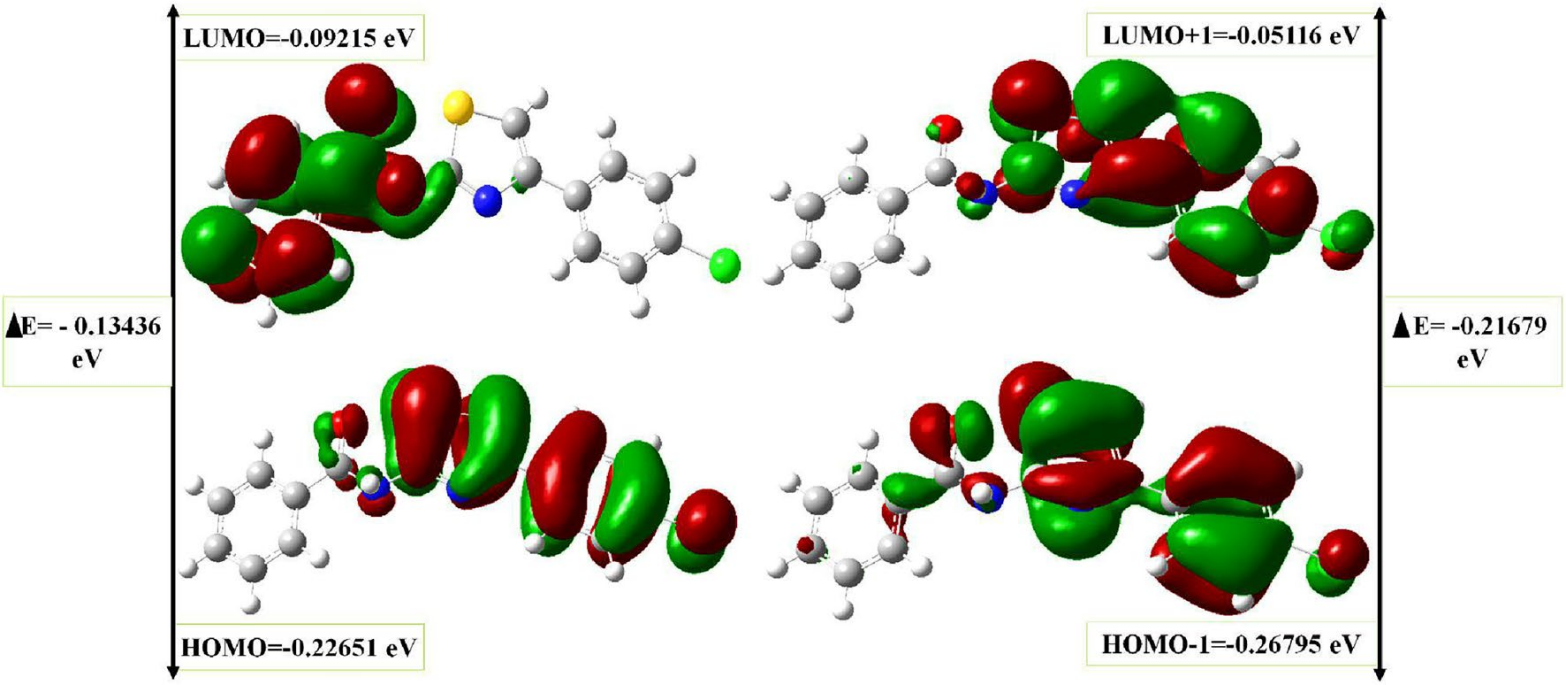
Distribution of molecular orbitals and their energy gaps at the HOMO, HOMO-1, LUMO, and LUMO+1 levels of Compound **4a**, determined using the B3LYP/631G(d, p) basis set. The HOMO and LUMO orbitals are predominantly anti-bonding orbitals, and the calculated HOMO–LUMO energy gap is 0.13436 eV.

The charge transfer interaction within the molecule could be explained by the energy gap of HOMO–LUMO. Results shown in Fig. 6 illustrates the molecular orbitals at various levels such as HOMO, HOMO-1, LUMO, LUMO+1, and their respective energy gaps determined with the B3LYP/631G(d, p) basis set. Our results showing The energy gap between HOMO and LUMO is 0.13436 eV while the energy gap of HOMO-1 and LUMO-1 orbitals was 0.10911 eV, these results attributed to that Compound **4a** is kinetically stable and is less polarizable, and less reactive compared to others.

### The effect of seed treatment with compounds 4a, 4b-h, and 4i on tomato seed quality parameters: experimental findings and implications for agricultural practices

The results showed that all chemicals used in the study were non-phytotoxic to tomato seeds. The analysis of seed germination and seedling vigor at the tested concentrations revealed that the seeds treated with compound 4a exhibited a slight reduction in germination percent and vigor index when compared to the control and other treatments. However, this reduction was not found to be phytotoxic. The study found that concentrations above 2% of the compounds showed phytotoxic effects under laboratory conditions. On the other hand, concentrations below 0.1% did not reduce wilt incidence under greenhouse conditions. Therefore, a concentration of 1% was selected for further in vivo studies in the greenhouse. Seeds treated with aliquots of methanol and made up the volume to 100 mL distilled water alone recorded a germination rate of 88% and a vigor index of 1186. In comparison, seeds treated with 1% of compound 4a, 4b, and 4d recorded germination rates of 90%, 89%, and 89%, respectively, with vigor index values of 1427, 1411, and 1386, respectively. Similarly, seed treatments with 4d and 4h at a 1% concentration recorded germination rates of 88% each, followed by vigor index values of 1358 and 1413, respectively, compared to the control.

### The study of antibacterial potential of seed treatment with compounds 4a, 4b–h, and 4i on bacterial wilt in tomato under greenhouse conditions

**Figure 7:**
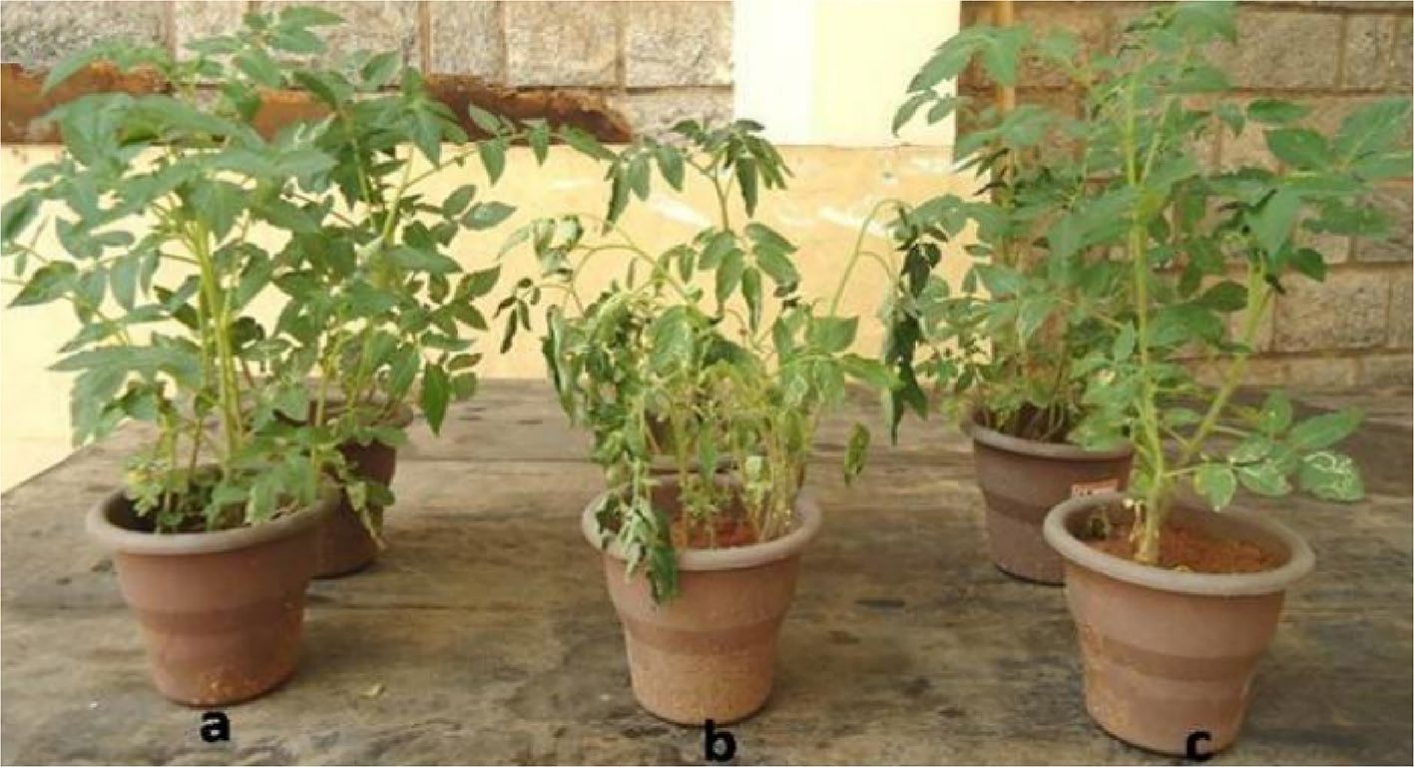
The effect of seed treatment with **4a** and **4f** nicotinamide analogs on the occur of bacterial wilt in tomato plants under greenhouse conditions. (**b**) Represents the control group of plants, where no seed treatment was applied. (**a**) and (**c**) Represent the treatment groups.

For evaluation the antimicrobial activity of the synthesised compounds (4a–i) under greenhouse conditions, we used ten treatments, included a control group, and the wilt incidence was measured as a percentage for each treatment. The control group represents tomato seeds without treatment which showed highest incidence of bacterial wilt (92.00 ± 0.82%). The other treatments showed varying levels of effectiveness in reducing the incidence of bacterial wilt. Treatment **4a** had the lowest incidence of wilt (28.70 ± 0.20%), indicating that it was the most effective treatment in reducing bacterial wilt in tomato plants under greenhouse conditions (Fig. 7). Treatment **4i** had the highest incidence of bacterial wilt (91.53 ± 0.47%), indicating that it was the least effective treatment (Table 4).

**Table 4:** Effect of seed treatment of tomato with **4a, 4b–h,** and **4i** on bacterial wilt incidence under greenhouse conditions.

Treatments	Wilt Incidence (%)
Control	92.00 ± 0.82^de^
**4a**	28.70 ± 0.20^a^
4b	81.10 ± 0.25^c^
4c	80.78 ± 0.83^c^
4d	72.63 ± 0.49^c^
4e	71.92 ± 0.49^c^
**4f**	39.43 ± 0.55^b^
4g	82.26 ± 0.90^cd^
4h	79.66 ± 0.57^c^
4i	91.53 ± 0.47^d^

Values are the means + SE of three replicates. Duncan’s multiple range tests (DMRT): Means sharing different alphabetical (a–e) superscripts in a columns significantly different (P < 0.05).

### Study the effect of compound 4a enhances plant defense enzymes boosts peroxidase (POX) and phenylalanine ammonia-lyase (PAL) against *R. solanacearum* in tomato plants

The objectives of the present study include evaluation the enhance of a synthesized compounds on the disease resistance of tomato plants by induction of defense enzymes which could have practical applications in the development of novel strategies to control bacterial wilt disease in plants^25^.

**Figure 8:**
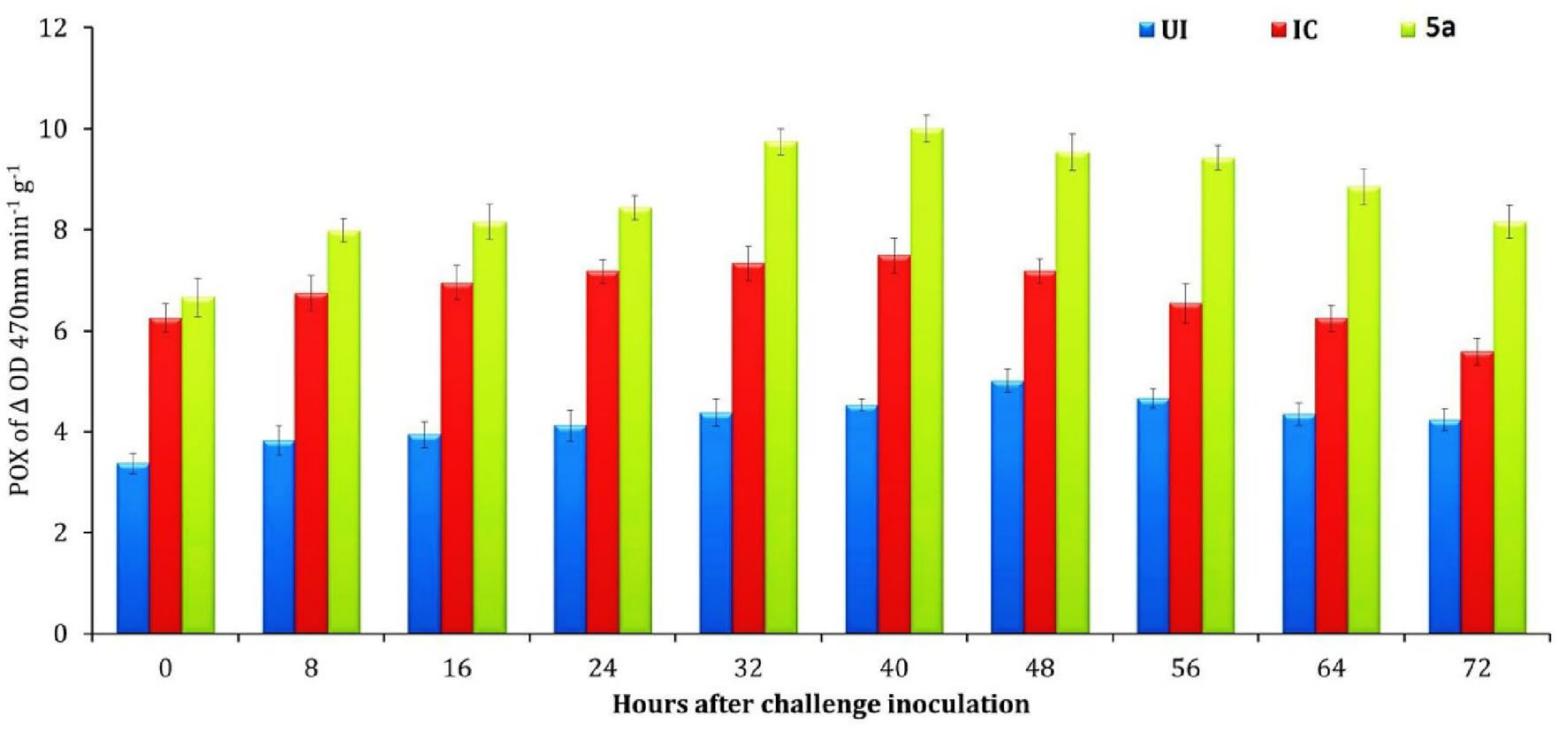
The impact of compounds on peroxidase activity in tomato seedlings. The mean of three replications is shown, and the bars indicate standard errors. The treatments are represented by **4a** for compound treatment, UI for the uninoculated control, and IC for the inoculated control (with compound challenged by *R. solanacea*-rum).

**Figure 9:**
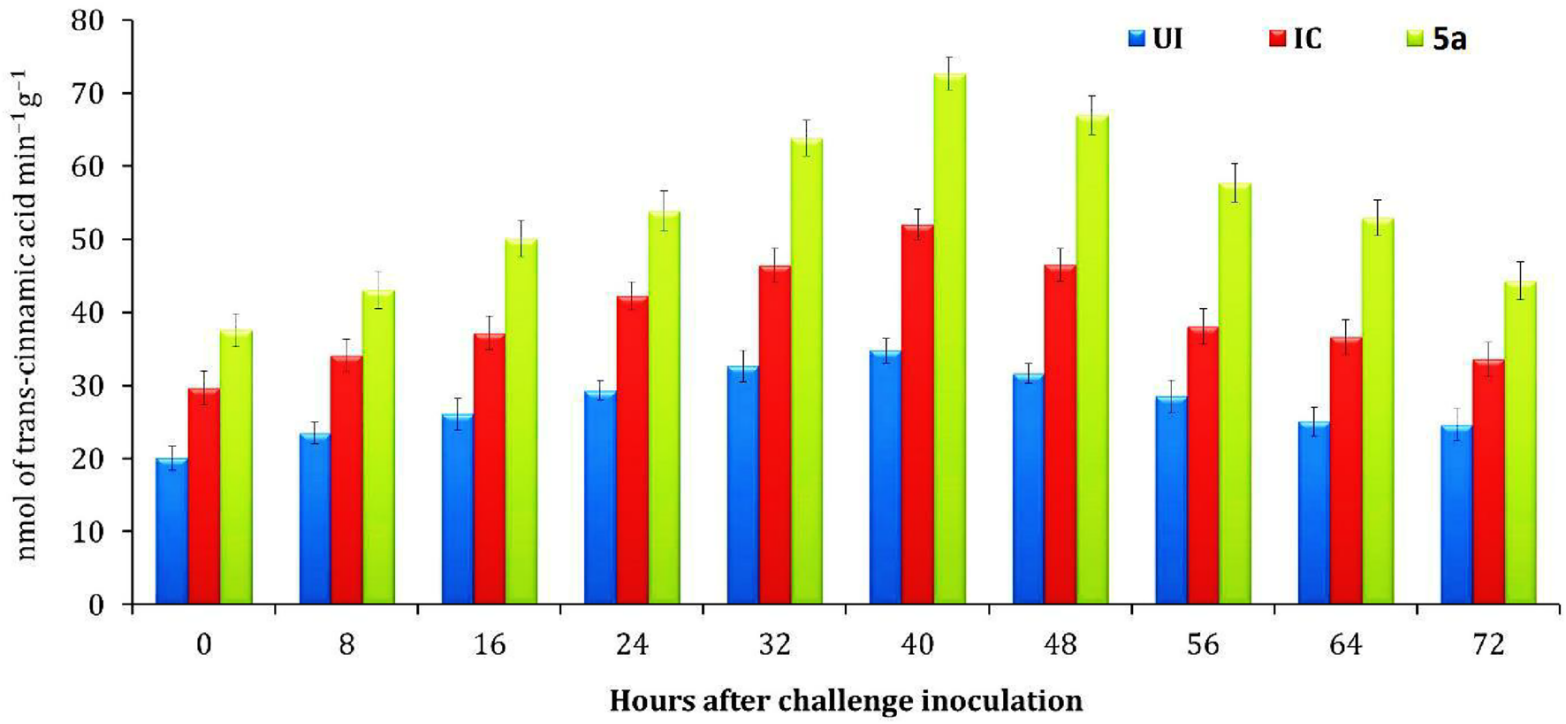
Effect of compounds on activity of phenylalanine ammonia-lyase in tomato seedlings. Values are the mean of three replications and bars represent standard errors. compound **4a** treatment; UI–Uninoculated control; IC–Inoculated control (compound challenged with *R. solanacearum*).

Treatment with the **4a** compound on tomato seedlings exhibited changes in the activities of two enzymes, peroxidase (POX) and phenylalanine ammonia lyase (PAL), at different periods. Specifically, the highest activities of these enzymes were observed at different times after treatment with the compound. POX and PAL are enzymes involved in the plant’s defense mechanism against pathogens. Peroxidases (POX) play a key role in the formation of reactive oxygen species (ROS), which are implicated in promoting or inhibiting cell growth and cell growth and elongation of the plant cell wall^26^. The results of the experiment showed that tomato seedlings inoculated with *R. solanacearum* and treated with the **4a** compound had a significantly increase in POX activity compared to the other treatments. The activity of POX enzyme reached its highest level in all treatments 40 h after the *R. solanacearum* inoculation and then slowly decreased. On the other hand, the PAL enzyme play important rule in the production some compounds like lignin and phytoalexins that protect the plant from infection^27^. Phenylalanine ammonia-lyase (PAL) is an enzyme involved in the biosynthesis of plant secondary metabolites. the activity of PAL was investigated in tomato seedlings in response to **4a** compound treatment and subsequent challenge with the plant pathogen *R. solanacearum*^28^. The results showed that PAL activity was significantly increased in the tomato seedlings treated with the **4a** compound and challenged with *R. solanacearum*, when compared to the untreated control. The highest PAL activity was reached maximum at 40 h after challenge inoculation, indicating that PAL was induced in response to the pathogen attack. Moreover, the PAL activity was found to be significantly higher in the compound **4a** treatment when compared to other treatments, suggesting that the compound 4a could enhance the plant's defense response against *R. solanacearum* by inducing PAL activity. This finding has important implications for the development of new plant disease control strategies using natural compounds, as it suggests that compounds like **4a** could be used to boost the plant's natural defense mechanisms and reduce reliance on synthetic pesticides. Overall, this study highlights the importance of PAL activity in the plant defense response and provides new insights into the potential use of natural compounds to enhance plant disease resistance as shown in Figs. 8 and 9.

### Seed treatment with compound 4a reduces bacterial wilt incidence in tomato plants under field conditions

The study conducted to investigate the effect of seed treatment with different compounds (**4a, 4b–h** and **4i**) on bacterial wilt incidence in tomato plants under field conditions. The experiment involved treating tomato seeds with compounds and then planting them in the field. The proportion (%) of disease incidence was calculated by dividing the number of wilted plants in a plot by the total number of plants in a plot, multiplied by 100. The control group was not treated with any compound. After the plants had grown for a certain period, the incidence of bacterial wilt was recorded as a percentage for each treatment. The results of reduces Bacterial Wilt under Field Conditions show highest incidence of bacterial wilt in control (90.58 ± 0.60%) while the treatment with 4a showed lowest incidence of bacterial wilt (32.37 ± 0.58%), which indicates that this compound was effective in controlling the disease. On the other hand, the treatments **4f** and **4b** also had significantly lower incidence of bacterial wilt compared to the control group and anther treatments (Table 5).

**Table 5:** Effect of seed treatment of tomato with 4a, 4b–h and 4i on bacterial wilt incidence under field conditions.

Treatments	Wilt incidence (%)
Control	90.58 ± 0.60^e^
**4a**	32.37 ± 0.58^a^
4b	84.83 ± 0.47^d^
4c	75.76 ± 0.56^c^
4d	74.32 ± 1.14^c^
4e	82.69 ± 0.43^cd^
**4f**	36.46 ± 0.53^b^
4g	72.32 ± 0.38^c^
4h	73.06 ± 0.79^c^
4i	82.65 ± 0.99^c^

Means of three replications, followed by the letters differently significant according to Duncan’s multiple range tests (DMRT). Values with different alphabetical (a–e) superscripts in a column significantly different (P ≤ 0.05). RS, *R. solanacearum*.

## Experimental

### Computational analysis of molecular structures studies

The installation of the Molecular Operating Environment (MOE) version 2015 for molecular modeling studies was executed utilizing the Windows 10 Pro operating system. The objective was to investigate the fucose-binding protein Monomeric *Ralstonia solanacearum* lectin (PDB ID: 4CSD)^29^ by retrieving it from the Protein Data Bank. The Discovery Studio 2019 client software was employed to examine the protein and eliminate non-interacting water molecules. To alter the protein’s partial charge, AutoDockTools-1.5.6 was utilized, followed by 3D protonation at a cutoff of 12.0. All non-standard ligands and residues were removed from the binding sites and the A chain of 4CSD was selected for docking studies and partial atomic charges were added to the protein structures and removed all water molecules from it. Proteins are saved in (Pdb) format; The atomic solubility coefficients were determined and finally converted into (Pdbqs) format.

The protein’s energy was subsequently reduced to a minimum through MOE with a gradient of (0.01 kJ mole^−1^) after adding additional hydrogen following standard geometry. The ChemDraw 18.1 software was utilized to sketch the ligands structure, and the Hamilton MMFF94 force field method was employed to correct the partial charges. 3D protonation and hydrogen addition were carried out according to standard geometry, and the energy of ligands was reduced to a minimum using Chem3D 18.1 with a gradient of (0.01 kJ mole^−1^) at a cutoff of 12. Initial simulated docking was conducted, followed by docking on specific active-site amino acids employing the sequence option. The best docking pose for each chemical structure was chosen from ths top ten. The highest pose score values from the series were used to conduct an analysis of docking and interaction. Furthermore, the software Discovery Studio was employed to create molecular modeling studies for the DNA-containing molecule 4a. The crystal structure of the B-DNA dodecamer d(CGCAAATTTCGC)2 (PDB ID: 1BNA) was retrieved from the Protein Data Bank to accomplish this.

### The frontier molecular orbitals: HOMO and LUMO investigations

Theoretical investigations in quantum chemistry, particularly focused on HOMO and LUMO studies, were executed using the advanced B3LYP/6-31G basis set via the Gaussian 09 program. To visualize the HOMO and LUMO orbitals, the state of the art visualization program, Gauss View 5.0^30^, was adeptly employed. These precise and intricate quantum chemical calculations and visualizations were carried out to delve deeper into the fundamental principles of molecular structures, reactivity, and electronic properties.

### Methods of chemical synthesis

**Scheme 1:**
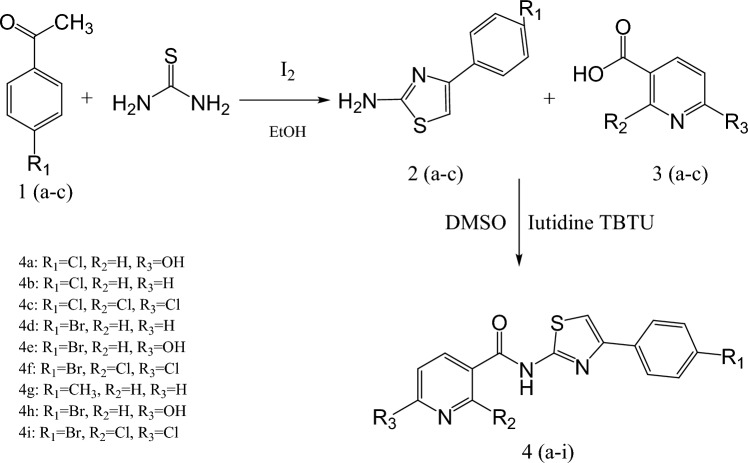
Synthesis of novel pyridine‐3‐ carboxamide derivatives 4a–i.

All the starting materials were commercially available, and it were obtained from Sigma Aldrich Company. Synthetic processes of the intermediates and target compounds were chemically synthesized the steps illustrated in Scheme 5. Characterization of new target compounds were performed by melting points determination by Stuart SMP30 (UK) and spectroscopic methods: Fourier-transform infrared spectroscopy (FTIR) Agilent Technologies’ state-of-the-art Cary 630 FTIR spectrometer, Nuclear magnetic resonance (NMR) at 400 MHz (VNMRS-400 Agilent-NMR spectrophotometer) and CHNS spectra analysis (Elementar Vario EL III elemental analyzer).Scheme 1Synthesis of novel pyridine‐3‐ carboxamide derivatives 4a–i.Scheme 1Synthesis of novel pyridine‐3‐ carboxamide derivatives 4a–i.Scheme 1Synthesis of novel pyridine‐3‐ carboxamide derivatives 4a–i.

### A comprehensive protocol for the production of 2-amino-4-phenylthiazoles (2a–c)

The inquiry entailed the fusion of novel 2-amino-4-phenyl thiazole analogues (2a–c) by well crushed the (0.0025 mol) of acetophenone derivatives (1a–c) and (0.003 mmol) thiourea in crucible. In 250 mL round bottom flask, the mixture was subjected to refluxing for a period of 16 h in the presence of (0.0062 mmol) of iodine and ethanol as solvent. After a white solid was precipitated, the solid was treated with sodium hydroxide solution to induce basification, after which recrystallization using ethanol yielded immaculate crystals, having a needle-like form as the ultimate product.

#### 2-Amino-4-(4-chlorophenyl)thiazole (2a)

Yield 82%; M.P. 169–171 °C. FT-IR (KBr, cm^−1^): 1698 (C=N), 3376 (N–H); 1H NMR (DMSO-d6): σ 7.11–7.93 (m, 5H, Ar–H), 7.45 (bs, 2H, NH2). LC–MS m/z 210 (M+), 212 (M+2). Anal. Calcd. for C_9_H_7_ClN_2_S: C, 51.31; H, 3.35. Found: C, 51.28; H, 3.29%.

#### 4-(P-tolyl)thiazol-2-amine (2b)

Yield 88%; M.P. 149–151 °C. FT-IR (KBr, cm^−1^): 1698 (C=N), 3376 (N–H); 1H NMR (DMSO-d6): σ 2.31 (s, 3H, CH_3_), 7.11–7.93 (m, 6H, Ar–H), 7.45 (bs, 2H, NH_2_). LC–MS m/z 191 (M+). Anal. Calcd. for C_10_H_10_N_2_S: C, 63.13; H, 5.30. Found: C, 63.11; H, 5.32%.

#### 2-Amino-4-(4-bromophenyl)thiazole (2c)

Yield 80%; M.P. 184–186 °C. FT-IR (KBr, cm^−1^): 1698 (C=N), 3376 (N–H); 1H NMR (DMSO-d6): σ 7.11–7.93 (m, 5H, Ar–H), 7.45 (bs, 2H, NH2). LC–MS m/z 255 (M+), 257 (M+2). Anal. Calcd. for C_9_H_7_BrN_2_S: C, 42.37; H, 2.77. Found: C, 42.34; H, 2.74%.

### The overarching synthetic methodology for *N*-(4-phenylthiazol-2-yl) nicotinamide derivatives (4a–i) is as follows

In this investigation, in (25 mL) a dry dichloromethane solution the (2 mmol) of nicotinic acid derivatives (3a–c) were dissolve and stirred at a temperature range of 25–30 °C. Afterward, the (3 mmol) of lutidine was added to the solution, followed by the addition (2 mmol) of substituted amino-4-phenyl-1,3-thiazoles (2a–c). The reaction blend was allowed to agitate at the same temperature. After 30, minutes the mixture was cold at 0–5 °C. (2 mmol) of TBTU was slowly added to cold mixture with kept the temperature below 5 °C. The reaction mixture was then mixed overnight using a mobile phase consisting of a blend of chloroform and methanol in a 9:1 proportion, ultimately resulting in a good yield of compounds 4a–i. The desired product was obtained by evaporating the solvent under low pressure, followed by quenching with crushed ice. The resultant solid was filtered, dried, and subsequently recrystallized from ethanol.

#### *N*-(4-(4-Chlorophenyl)thiazol-2-yl)-6-hydroxynicotinamide (4a)

Yield 75%; M.P. 200–202 °C; FT-IR (KBr, cm^−1^): 1445 (C=N, nicotinamide), 1675 (C=O), 1698 (C=N, thiazol), 3176 (N–H); 1H NMR (400 MHz, DMSO): σ 6.75–8.22 (m, 8H, Ar–H), 11.65 (S, 1H, OH), 12.76 (s, 1H, NH); 13C NMR (400 MHz, DMSO): σ 106.01, 112.73, 115.74, 129.71, 131.11, 134.37, 136.12, 145.57, 150,21, 154.66, 164.77; LC–MS m/z 332 (M+), 334 (M+2). Anal. Calcd. For C_15_H_10_ClN_3_O_2_S: C, 54.30; H, 3.04. Found: C, 54.28; H, 3.01%.

#### *N*-(4-(4-Chlorophenyl)thiazol-2-yl)nicotinamide (4b)

Yield 83%; M.P. 155–157 °C; FT-IR (KBr, cm^−1^): 1445 (C=N, nicotinamide), 1675 (C=O), 1698 (C=N, thiazol), 3176 (N–H); 1H NMR (400 MHz, DMSO): σ 6.98–7.87 (m, 9H, Ar–H), 12.46 (s, 1H, NH); 13C NMR (400 MHz, DMSO): σ 101.02, 125.13, 129.34, 132.01, 135.01, 148.77, 150.22, 164.77; LC–MS m/z 316 (M+), 318 (M+2). Anal. Calcd. For C_15_H_10_ClN_3_OS: C, 57.05; H, 3.19. Found: C, 57.01; H, 3.16%.

#### 2,6-Dichloro-*N*-(4-(4-chlorophenyl)thiazol-2-yl)nicotinamide (4c).

Yield 75%; M.P. 186–188 °C; FT-IR (KBr, cm^−1^): 1445 (C=N, nicotinamide), 1675 (C=O), 1698 (C=N, thiazol), 3176 (N–H); 1H NMR (400 MHz, DMSO): σ 7.52–8.78 (m, 7H, Ar–H), 12.76 (s, 1H, NH); 13C NMR (400 MHz, DMSO): σ 105.01, 124.43, 129.31, 131.11, 134.37, 143.62, 150,11, 152.26, 164.77; LC–MS m/z 384 (M+), 386 (M+2), 388 (M+4). Anal. Calcd. For C_15_H_8_Cl_3_N_3_OS: C, 46.84; H, 2.10. Found: C, 46.82; H, 2.07%.

#### *N*-(4-(4-Bromophenyl)thiazol-2-yl)nicotinamide (4d)

Yield 83%; M.P. 211–213 °C; FT-IR (KBr, cm^−1^): 1445 (C=N, nicotinamide), 1675 (C=O), 1698 (C=N, thiazol), 3176 (N–H); 1H NMR (400 MHz, DMSO): σ 7.52–9.18 (m, 9H, Ar–H), 12.76 (s, 1H, NH); 13C NMR (400 MHz, DMSO): σ 105.02, 123.13, 125.14, 128.33, 130.71, 132.11, 135.33, 148.77, 150.22, 164.77; LC–MS m/z 361 (M+), 363 (M+2). Anal. Calcd. For C_15_H_10_BrN_3_OS: C, 50.01; H, 2.80. Found: C, 50.03; H, 2.82%.

#### *N*-(4-(4-Bromophenyl)thiazol-2-yl)-6-hydroxynicotinamide (4e)

Yield 75%; M.P. 222-224 °C; FT-IR (KBr, cm^−1^): 1445 (C=N, nicotinamide), 1675 (C=O), 1698 (C=N, thiazol), 3176 (N–H); 1H NMR (400 MHz, DMSO): σ 6.95–8.22 (m, 8H, Ar–H), 11.65 (S, 1H, OH), 12.76 (s, 1H, NH); 13C NMR (400 MHz, DMSO): σ 105.01, 112.73, 115.74, 123.31, 128.32, 132.11, 136.12, 145.57, 150,21, 154.66, 164.77; LC–MS m/z 336 (M+), 338 (M+2). Anal. Cal. For C_15_H_10_BrN_3_O_2_S: C, 47.89; H, 2.68. Found: C, 47.91; H, 2.71%.

#### *N*-(4-(4-Bromophenyl)thiazol-2-yl)-2,6-dichloronicotinamide (4f)

Yield 75%; M.P. 244–246 °C. FT-IR (KBr, cm^−1^): 1445 (C=N, nicotinamide), 1675 (C=O), 1698 (C=N, thiazol), 3176 (N–H); 1H NMR (400 MHz, DMSO): σ 7.52–8.78 (m, 7H, Ar–H), 12.76 (s, 1H, NH); 13C NMR (400 MHz, DMSO): σ 105.01, 123.20, 124.43, 128.31, 132.11, 134.37, 143.62, 150,11, 152.26, 164.77; LC–MS m/z 429 (M+), 431 (M+2), 433 (M+4). Anal. Cal. For C_15_H_8_BrCl_2_N_3_OS: C, 41.99; H, 1.88. Found: C, 41.97; H, 1.85%.

#### *N*-(4-(P-tolyl)thiazol-2-yl)nicotinamide (4g)

Yield 83%; M.P. 199–201 ^o^C. FT-IR (KBr, cm^−1^): 1445 (C=N, nicotinamide), 1675 (C=O), 1698 (C=N, thiazol), 3176 (N–H); 1H NMR (400 MHz, DMSO): σ 2.34 (s, 3H, CH3), 7.24–9.18 (m, 9H, Ar–H), 12.76 (s, 1H, NH); 13C NMR (400 MHz, DMSO): σ 21.31, 105.02, 125.73, 129.54, 130.71, 131.71, 135.37, 148.77, 150.22, 164.77; LC–MS m/z 396 (M+). Anal. Cal. For C_16_H_13_N_3_OS C, 65.07; H, 4.44. Found: C, 65.04; H, 4.41%.

#### *N*-(4-(4-Bromophenyl)thiazol-2-yl)-6-hydroxynicotinamide (4h)

Yield 75%; M.P. 233–235 °C. FT-IR (KBr, cm^−1^): 1445 (C=N, nicotinamide), 1675 (C=O), 1698 (C=N, thiazol), 3176 (N–H); 1H NMR (400 MHz, DMSO): σ 6.75–8.22 (m, 8H, Ar–H), 11.65 (S, 1H, OH), 12.76 (s, 1H, NH); 13C NMR (400 MHz, DMSO): σ 106.01, 112.73, 115.74, 123.21, 128.31, 132.17, 136.12, 145.57, 150,21, 154.66, 164.77; LC–MS m/z 377 (M+), 379 (M+2). Anal. Calcd. For C_15_H_10_BrN_3_O_2_S: C, 47.89; H, 2.68. Found: C, 47.86; H, 2.66%.

#### N-(4-(4-Bromophenyl)thiazol-2-yl)-2,6-dichloronicotinamide (4i)

Yield 75%; M.P. 210–212 °C. FT-IR (KBr, cm^−1^): 1445 (C = N, nicotinamide), 1675 (C=O), 1698 (C=N, thiazol), 3176 (N–H); 1H NMR (400 MHz, DMSO): σ 7.52–8.78 (m, 7H, Ar–H), 12.76 (s, 1H, NH); 13C NMR (400 MHz, DMSO): σ 105.01, 124.43, 128.31, 132.11, 133.87, 143.62, 150,11, 152.26, 164.77; LC–MS m/z 429 (M+), 431 (M+2), 433 (M+4). Anal. Calcd. For C_15_H_8_BrCl_2_N_3_OS: C, 41.99; H, 1.88. Found: C, 41.97; H, 1.86%.

### Elucidation and characterization of R. solanacearum through a systematic investigative approach

#### Sample collection

In this study, tomato plants suspected of being infected with bacterial wilt were carefully collected from tomato fields. The experiment was conducted during the 2018 tomato growing season on large agricultural plot 1 km belonging to agricultural college of University of Mysore in Bhoomishettihalli, Karnataka, India. The seeds were treated with non-phytotoxic doses of the test chemicals (1%) for a period of 6 h prior to sowing in pots filled with sterilized potting soil consisting of a 2:1:1 ratio of soil, sand, and coconut pith compost. The seedlings were then transferred to experimental plots with a spacing of 60–90 cm, and each experimental plot measured 25 m^2^ and contained 14 rows with 80–100 plants per row, with a 50 cm gap between each row. The experiment was repeated three times simultaneously in three separate experimental plots in the field. The seedlings were watered with daily drip irrigation and received farmyard manure (FYM) at a rate of 2.8 kg m^−2^ and vermicompost at a rate of 0.5 kg m^−2^. 2 weeks after transplantation, a 48-h *R. solanacearum* suspension was drenched into the soil to serve as a challenge.

#### Isolation of *R. solanacearum*

The small pieces of suspected plant were sterilized with a 1% NaOCl solution for 2 min, followed by dilution with DW and blot dried. Then the plant sections were then plated onto 2, 3, 5-triphenyl tetrazolium chloride (Kelman’s TZC agar) medium. The plant sections were then plated onto 2, 3, 5 triphenyl tetrazolium chloride (Kelman’s TZC agar) medium. The plates were incubated at 28 °C ± 2 °C for 24–48 h. The virulent colonies in the medium characterized by dull white color, fluidal, irregularly round with light pink centers were further streaked on TZC medium to get pure colonies of the *R. solanacearum* bacteria.

#### Identification of isolates

The identification of the selected R. solanacearum isolate was then confirmed through the use of molecular methods, specifically 16S rRNA sequencing. To carry out the 16S rRNA sequencing, the 16S rRNA gene was first amplified by PCR using universal primers 8F (5′-AGAGTTTGATCCTGGCTCAG-3') and 806R (5′-GGACTACCAGGGTATCTAAT-3′) as previously described by Lane (1991). A (100 ng) of template was added to a reaction mix containing (20 mM) of Tris–HCl (pH 8.4), (50 mM) KCl, (1.5 mM) MgCl_2_, (2 μL) of each dNTPs, (0.4 μM) of each primer and (2.5 U μL^−1^) Taq polymerase to complete a final volume of 50 μL. The PCR conditions were: initial denaturation of 2 min at 94 °C, followed by 35 cycles of 1 min at 94 °C, 1.5 min at 55 °C and 1 min at 72 °C. The reaction product was visualized on a (1%) agarose gel under UV light after ethidium bromide staining.

The resulting PCR product was then sequenced, and the obtained sequences were analyzed using BLAST. Finally, the sequences obtained were deposited in the NCBI GenBank with the accession number KF924743. This process allowed for the accurate identification of the bacterial strain responsible for the tomato plant infection, providing valuable information for disease management and prevention efforts.

### Development of microbial culture for inoculation

A stock suspension of *Ralstonia solanacearum* was used to inoculate a broth of casamino acid peptone glucose (CPG), which was prepared by mix 0.1% casamino acid, 1% peptone, 0.5% glucose, CPG supplemented with 0.005% (w/v) 2, 3, 5-triphenyltetrazolium chloride (TZC) and M9 media, which contains 20% M9 salts (5×), 2% glucose, 0.2% MgSO_4_, 0.01% CaCl_2_. The inoculated broth was then incubated at a temperature of 28 °C on a rotary shaker at 150 rpm for a period of 48 h. Following the incubation period, the culture broth was subjected to a (10 min) centrifugation at a speed of 12,000 rpm and a temperature of 10 °C. The bacterial pellet was then re-suspended in sterile distilled water and the final concentration of the suspension was set to 1108 cfu mL^−1^ by spectro-photometrically correcting to O.D. 600 nm = 0.1. This process allowed for the preparation of a highly concentrated bacterial suspension, which was essential for subsequent experiments.

### Influence of priming with 4a, 4b-h, and 4i on tomato seed quality metrics

The present study was performed to evaluate the effects of pyridine-3-carboxamide analogs on tomato seed germination and seedling vigor in laboratory conditions. before the testing, non-phytotoxic doses of the compounds were determined through preliminary investigations of seed germination and seedling vigor. For the experiment, Abhinav cv seeds were treated with the test chemicals, which were first dissolved in methanol aliquots and then diluted with water to final doses of 0.001%, 0.01%, 0.1%, and 1%. After a six-hour soaking period, the seeds were subjected to the usual blotter procedure (ISTA 2005) by plating 25 seeds on three-layered moist blotter discs in 9-cm-diameter plastic Petri plates. The plates were then incubated at a temperature of 24–1 °C for a period of 12 days, with controls being treated with distilled water. The experiment was repeated three times with four replicates of 100 seeds for each treatment. After 12 days of incubation, the percentage of germination, root and branch lengths, and seedling vigor were measured and estimated using a formula^31^.

$$\begin{aligned} \% {\text{Seed Germination}} & \, = {\text{ Number of seeds germinated}}/{\text{Total Number of seeds plated}} \times { 1}00{\text{ Vigor Index}} \\ & = \, \left( {{\text{Mean root length }} + {\text{ Mean shoot length}}} \right) \, \times {\text{ Germination percentage}} \\ \end{aligned}$$

The results were then analyzed to determine the impact of the tested compounds on tomato seed quality parameters. Overall, this study provides valuable insights into the effects of pyridine-3-carboxamide analogs on tomato seed germination and seedling vigor, with the potential to enhance plant immunity against bacterial blight pathogens. The use of a rigorous experimental design allowed for accurate and reliable results, which can contribute to the development of effective disease management strategies in agriculture.

### Unveiling the influence of 4a, 4b–h, and 4i seed treatment on the development of bacterial wilt in tomatoes grown under greenhouse conditions

The current study aimed to investigate the impact of seed treatment with 4a, 4b–h, and 4i on the incidence of bacterial wilt in tomato plants under greenhouse conditions. To carry out the experiment, tomato seeds were treated with non-phytotoxic concentrations (1%) of the test chemicals for a period of 6 h prior to sowing in portrays filled with sterilized potting soil consisting of a 2:1:1 ratio of soil, sand, and coconut pith compost. The seedlings were then watered regularly in preparation for transplantation. Healthy tomato seedlings that were 21 days old were transplanted into earthen pots with a diameter of 30 cm and a depth of approximately 1.5 cm. The pots were filled with a sterilized mixture of red soil, sand, and farmyard manure (FYM) in a 2:1:1 w/w ratio, with three seedlings per pot. The seedlings were then challenge-inoculated by trimming the lower roots with sterilized scissors and dipping them in a *R. solanacearum* suspension of 1 × 108 CFU mL^−1^ for a period of 1 h. After inoculation, the seedlings were kept in the greenhouse at a daily temperature range of 18–28 °C and a relative humidity of 60–96%. Each treatment consisted of 20 pots with three seedlings per pot, with non-inoculated seedlings serving as controls. The experiment included 60 plants per treatment with three replications, and the pots were set up in a randomized design. The plants were watered regularly, and daily observations were made to monitor the symptoms of wilt disease. At the end of 30 days after pathogen inoculation, the percentage of disease incidence was noted. This allowed for the determination of the impact of seed treatment with **4a, 4b–h**, and **4i** on the incidence of bacterial wilt in tomato plants under greenhouse conditions. The use of a rigorous experimental design and careful monitoring of the plants allowed for accurate and reliable results, which can contribute to the development of effective disease management strategies in agriculture.

### Optimizing sample collection for enzyme analysis: strategies and techniques

For enzyme analysis, leaf samples were collected from plants that were treated with compound 4a and plants that were not treated at various time points, including 0, 8, 16, 24, 32, 40, 48, 56, 64, and 72 h after the challenge inoculation. These samples were then kept at a temperature of – 80 °C until they were used for further analysis. To prepare the samples for analysis, (1 g) of tomato leaf tissues were homogenized using liquid nitrogen in a pre-chilled mortar and pestle. The homogenized leaf tissues were then mixed with 2 mL of 0.1 M sodium phosphate buffer solution at a pH of 7.0 and kept at a temperature of 4 °C. The resulting homogenate was centrifuged at a speed of 12,000 rpm for a duration of (20 min), and the supernatant was used as a crude extract for the subsequent analysis of peroxidase (POX) and phenylalanine ammonia-lyase (PAL) assays. The POX activity was expressed as a change in absorbance per minute per gram of fresh leaves, while the PAL activity was expressed as the amount of enzyme that produces nmol of trans-cinnamic acid per minute per gram of fresh leaf tissues. The total phenolic activity was expressed as µg of catechol per gram of plant tissue. Additionally, protein estimations of all the enzyme extracts were carried out using Lowry’s method, with bovine serum albumin serving as a standard. Overall, this study provides valuable insights into the impact of compound 4a on the activity of POX and PAL enzymes in tomato plants. The use of rigorous sample collection and analysis techniques allowed for accurate and reliable results, which can contribute to the development of effective disease management strategies in agriculture.

### Evaluating the influence of 4a, 4b–h, and 4i seed treatment on bacterial wilt development in tomatoes cultivated under field conditions

To investigate the effect of seed treatment with 4a, 4b–h, and 4i on the incidence of bacterial wilt in tomato plants under field conditions, a randomized methodology was employed. The chemicals that demonstrated a preventive effect in greenhouse tests were advanced to the field testing stage. The field experiment was carried out during the 2018 tomato growing season on agricultural plots belonging to a farmer in Bhoomishettihalli (BH) located near Chintamani, Karnataka, India. Abhinav seeds were treated with non-phytotoxic doses of the test chemicals (1%) for a period of 6 h prior to sowing in pots filled with sterilized potting soil consisting of a 2:1:1 ratio of soil, sand, and coconut pith compost. The seedlings, which were four weeks old, were then transferred from pots to experimental plots with a spacing of 60–90 cm. Each experimental plot measured 25 m^2^ and contained 14 rows with 80–100 plants per row, with a 50 cm gap between each row. The experiment was repeated three times simultaneously in three separate experimental plots in the field. The seedlings were watered with daily drip irrigation and received farmyard manure (FYM) at a rate of 2.8 kg m^−2^ and vermicompost at a rate of 0.5 kg m^−2^. Two weeks after transplantation, a 48-h *R. solanacearum* suspension was drenched into the soil to serve as a challenge. The proportion (%) of disease incidence was calculated by dividing the number of wilted plants in a plot by the total number of plants in a plot, multiplied by 100. The results of the study are presented in Table 5, which provides the percentage of wilt incidence for each treatment. The experiment was designed in a randomized manner, and the results were analyzed using Duncan’s multiple range tests (DMRT). The results showed that the control group had the highest incidence of bacterial wilt at 90.58 ± 0.60%, indicating that the disease was able to spread easily in the untreated plants.

The treatment with **4a** resulted in the lowest incidence of bacterial wilt at 32.37 ± 0.58%, followed by 4f and 4b, which also had significantly lower incidence of bacterial wilt compared to the control group, with percentages of 36.46 ± 0.53% and 84.83 ± 0.47%, respectively. Treatments **4c, 4d, 4e, 4g, 4h** and **4i** showed moderate levels of bacterial wilt incidence, with percentages ranging from 72.32 ± 0.38% to 82.69 ± 0.43%. These treatments were less effective in controlling the disease compared to 4a and 4f but still had a significant effect compared to the control group. So, the study provides valuable insights into the impact of seed treatment with different compounds on the incidence of bacterial wilt in tomato plants under field conditions.

### Molecular docking

Molecular docking was conducted for the protein–compound complexs to determine the binding affinity of synthesis compound agents *R. solanacearum* protien. We used the Auto dock program to perform the resulting best-docked protein–ligand complexes. Before the molecular docking, the ligands and the target proteins were prepared. Firstly, the structures were optimized adding the required changes such as Kollman and Gasteiger charges. Polar hydrogen atoms were also added to the protein structures.

### Data analysis with statistical methods: techniques and approaches

The arcsine transformation and analysis of variance were performed using JMP software by SAS Institute Inc., situated in Cary, NC. The statistical significance of the impact of the PGPR treatments was established by the F-value (P = 0.05), and Duncan's multiple range test (DMRT) was used to segregate treatment means.

### Ethics approval and consent to participate

Tomato plant and seed were collected from agricultural college of University of Mysore in Bhoomishettihalli, Karnataka, India and were identified by professor Yasser Hussein Issa Mohammed. The methods involved in this study were carried out in compliance with local and national regulations.

### Human or animal subjects

As this is research article, it does not involve any animal or human study.

## Conclusion

**Figure 10:**
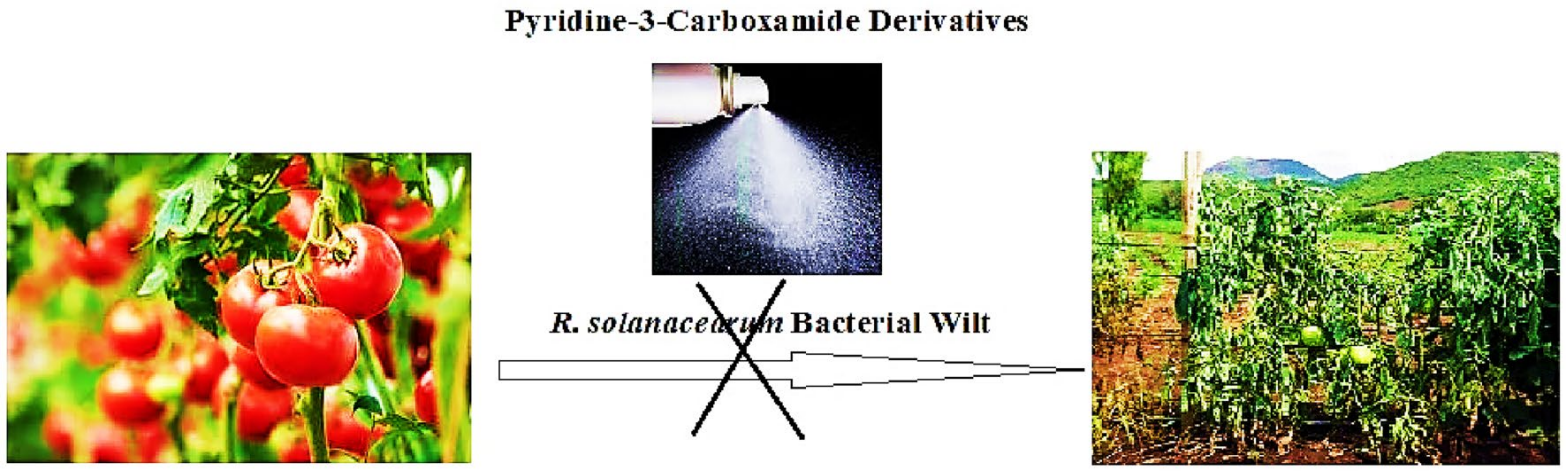
The green miracle: unraveling the secrets of compound 4a in tomato protection.

This study presents a novel series of nicotinamide analogs that possess potent antibacterial activity against *R. solanacearum*, a bacterial wilt pathogen, and promote the growth of tomato plants. The results of this study suggest that compound **4a** had ability to reduce the severity of the bacterial wilt pathogen disease in tomatoes both in a screen house and in the field. The compound **4a** showed a correlation with enzyme data, which shows that it could be a powerful antibacterial wilt pathogen treatment for tomato plants that have been infected. The molecular docking experiments demonstrated that compound **4a** had a significant effect on tomato resistance against the bacterial wilt pathogen *Ralstonia solanacearum*, exhibiting strong binding energy. In vitro studies were conducted to test the antagonistic properties of compounds 4a–i against *R. solanacearum*, the causal agent of bacterial disease in tomatoes. Furthermore, a field experiment was conducted to evaluate the efficacy of compounds 4a-i against the bacterial wilt pathogen *R. solanacearum*. The results revealed that the substituted groups, including one chloro group at the para position in rings C and one hydroxy group at the ortho position in rings A, as found in compound **4a**, are crucially important as they elicit target-specific action against the bacterial wilt pathogen. Additionally, compound **4a** demonstrated high effectiveness in reducing the incidence of bacterial wilt in tomato plants as represented in Fig. 10.

### Supplementary Information

Supplementary Table S1.

## Data Availability

The data supporting this study’s findings are available at the request of the corresponding author.

## References

[1] Ajilogba CF, Babalola OO (2013). Integrated management strategies for tomato Fusarium wilt. Biocontrol Sci..

[2] Hong JK, Jang SJ, Lee YH (2018). Reduced bacterial wilt in tomato plants by bactericidal peroxyacetic acid mixture treatment. Plant Pathol. J..

[3] Umamaheswari A, Puratchikody A, Hari N (2019). Synthesis and investigation of therapeutic potential of isoform-specific HDAC8 inhibitors for the treatment of cutaneous t cell lymphoma. Anti-Cancer Agents Med. Chem..

[4] Kim SG (2016). Evaluation of resistance to *Ralstonia solanacearum* in tomato genetic resources at seedling stage. Plant Pathol. J..

[5] Brown DG, Allen C (2004). *Ralstonia solanacearum* genes induced during growth in tomato: An inside view of bacterial wilt. Mol. Microbiol..

[6] Sun Y (2023). Biocontrol of bacterial wilt disease in tomato using *Bacillus subtilis* strain R31. Front. Microbiol..

[7] Karpina V (2019). The synthesis and biological assessment of [[1, 2, 4] triazolo [4, 3-a] pyridine-3-yl] acetamides with an 1, 2, 4-oxadiazol cycle in positions 6, 7 and 8. J. Org. Pharm. Chem..

[8] Cañedo-Castro B, Piñón-Gimate A, Carrillo S, Ramos D, Casas-Valdez M (2019). Prebiotic effect of *Ulva rigida* meal on the intestinal integrity and serum cholesterol and triglyceride content in broilers. J. Appl. Phycol..

[9] Wicker E, Grassart L, Coranson-Beaudu R, Mian D, Guilbaud C, Fegan M, Prior P (2007). *Ralstonia solanacearum* strains from Martinique (French West Indies) exhibiting a new pathogenic potential. Appl. Environ. Microbiol..

[10] Kumar A, Prameela TP, Suseelabhai R, Siljo A, Anandaraj M, Vinatzer BA (2014). Host specificity and genetic diversity of race 4 strains of R alstonia solanacearum. Plant Pathol..

[11] Mohammed YHE (2018). The Novel 4-Phenyl-2-Phenoxyacetamide Thiazoles modulates the tumor hypoxia leading to the crackdown of neoangiogenesis and evoking the cell death. Eur. J. Med. Chem..

[12] Kemboi VJ, Kipkoech C, Njire M, Were S, Lagat MK, Ndwiga F, Wesonga JM, Tanga CM (2022). Biocontrol potential of chitin and chitosan extracted from black soldier fly pupal exuviae against bacterial wilt of tomato. Microorganisms.

[13] Hong JK, Jang SJ, Lee YH, Jo YS, Yun JG, Jo H, Park CJ, Kim HJ (2018). Reduced bacterial wilt in tomato plants by bactericidal peroxyacetic acid mixture treatment. Plant Pathol. J..

[14] Chen J, Yu Y, Li S, Ding W (2016). Resveratrol and coumarin: Novel agricultural antibacterial agent against *Ralstonia solanacearum* in vitro and in vivo. Mol. Basel Switzerl..

[15] Yuliar-Nion YA, Toyota K (2015). Recent trends in control methods for bacterial wilt diseases caused by *Ralstonia solanacearum*. Microbes Env..

[16] Mann A, Nehra K, Rana JS, Dahiya T (2021). Antibiotic resistance in agriculture: Perspectives on upcoming strategies to overcome upsurge in resistance. Curr. Res. Microbial Sci..

[17] Wu J, Kang S, Song B, Hu D, He M, Jin L, Yang S (2012). Synthesis and antibacterial activity against *Ralstonia solanacearum* for novel hydrazone derivatives containing a pyridine moiety. Chem. Central J..

[18] Agu PC, Afiukwa CA, Orji OU, Ezeh EM, Ofoke IH, Ogbu CO, Ugwuja EI, Aja PM (2023). Molecular docking as a tool for the discovery of molecular targets of nutraceuticals in diseases management. Sci. Rep..

[19] Aber QAH, Shentaif AH, Almajidi M (2023). Synthesis, structure, and in vitro pharmacological evaluation of some new pyrimidine-2-sulfonamide derivatives and their molecular docking studies on human estrogen receptor alpha and CDK2/Cyclin proteins. Russ. J. Bioorg. Chem..

[20] Bartzatt RL (2004). Evaluation of pyridine-3-carboxylic acid as a drug carrier by utilizing multivariate methods, structure property correlations, and pattern recognition techniques. Recept. Channels.

[21] Ling Y, Hao ZY, Liang D, Zhang CL, Liu YF, Wang Y (2021). The expanding role of pyridine and dihydropyridine scaffolds in drug design. Drug Design Dev. Therapy.

[22] Wang S, Yuan XH, Wang SQ, Zhao W, Chen XB, Yu B (2021). FDA-approved pyrimidine-fused bicyclic heterocycles for cancer therapy: Synthesis and clinical application. Eur. J. Med. Chem..

[23] Edraki N, Mehdipour AR, Khoshneviszadeh M, Miri R (2009). Dihydropyridines: Evaluation of their current and future pharmacological applications. Drug Discov. Today.

[24] Marinescu M, Popa CV (2022). Pyridine compounds with antimicrobial and antiviral activities. Int. J. Mol. Sci..

[25] Chakraborty N, Chandra S, Acharya K (2017). Biochemical basis of improvement of defense in tomato plant against Fusarium wilt by CaCl2. Physiol. Mol. Biol. Plants.

[26] Kawano T (2003). Roles of the reactive oxygen species-generating peroxidase reactions in plant defense and growth induction. Plant Cell Rep..

[27] Lavanya SN, Niranjan-Raj S, Jadimurthy R, Sudarsan S, Srivastava R, Tarasatyavati C, Rajashekara H, Gupta VK, Nayaka SC (2022). Immunity elicitors for induced resistance against the downy mildew pathogen in pearl millet. Sci. Rep..

[28] Singh K, Kumar S, Rani A, Gulati A, Ahuja PS (2009). Phenylalanine ammonia-lyase (PAL) and cinnamate 4-hydroxylase (C4H) and catechins (flavan-3-ols) accumulation in tea. Funct. Integr. Genom..

[29] Kostlánová N, Mitchell EP, Lortat-Jacob H, Oscarson S, Lahmann M, Gilboa-Garber N, Chambat G, Wimmerová M, Imberty A (2005). The fucose-binding lectin from *Ralstonia solanacearum*. A new type of beta-propeller architecture formed by oligomerization and interacting with fucoside, fucosyllactose, and plant xyloglucan. J. Biol. Chem..

[30] La-Porta FA, Ramalho TC, Santiago RT, Rocha MV, Da-Cunha EF (2011). Orbital signatures as a descriptor of regioselectivity and chemical reactivity: The role of the frontier orbitals on 1, 3-dipolar cycloadditions. J. Phys. Chem. A.

[31] Adesiyan IM, Bisi-Johnson MA, Ogunfowokan AO, Okoh AI (2019). Incidence and antimicrobial susceptibility fingerprints of Plesiomonas shigelliodes isolates in water samples collected from some freshwater resources in Southwest Nigeria. Sci. Total Environ..

