# Supplementary material for: Synthesis and evaluation of pyridine-3-carboxamide analogs as effective agents against bacterial wilt in tomatoes
Source: Sci Rep. 2024 May 15;14:11118. doi: 10.1038/s41598-024-59609-z (PMC11096348; doi:10.1038/s41598-024-59609-z)
Supplement: Supplementary file 1 — Supplementary Table S1. [file 41598_2024_59609_MOESM1_ESM.docx]

**Table S1.** Structure-Activity Relationship (SAR) of compounds **4a-i** with antibacterial (PDB:4CSD)

| **Name of compound** |  |  | **Binding**  **energy**  **(kJ mol^-1^)** | | | **Ligand**  **efficiency** | **Inhibition**  **constant** | **vdW+H-bond**  **+desolv energy** | **No. of**  **H- bonds** | **Amino acid**  **Residues of docked domains** | | **Bonding residues** | **Bond**  **length**  **(Å)** |  |
| --- | --- | --- | --- | --- | --- | --- | --- | --- | --- | --- | --- | --- | --- | --- |
|  |  |  |  |  |  |  |  |  |  |  |  |  |  |  |
|  |  |  |  |  |  |  |  |  |  |  |  |  |  |  |
|  |  |  |  |  |  |  |  |  |  |  |  |  |  |  |
|  |  |  |  |  |  |  |  |  |  |  |  |  |  |  |
|  |  |  |  | | |  |  |  |  | Met 0,Cys 30, | | 4csd:A : THR 51: HG1: 5B: 1: N: 5B: 1:H: 4csd:A : SER 52:O: 4csd:A : | 1.937 |  |
|  |  |  |  | | |  |  |  |  | Asn 42,Thr 67, | | ALA 7: HN: 5B: 1: S | 2.076 |  |
| 4a |  |  | -8.910 | | | -0.38 | 683.25 | -9.32 | 4 | Cy s 75, Ser | 88, | 5B: 1:H : 4csd:A: ALA7:O | 2.231 |  |
|  |  |  |  | | |  |  |  |  | Cys 121,Gly159 | |  | 2.189 |  |
|  |  |  |  | | |  |  |  |  | Cys 166,Cys 21 | |  |  |  |
| 4b |  |  | -6.884 | | | -0.13 | 18.22 | -6.98 | 2 | Cys 257, Thr 271 |  | 4csd:A : ASN 138: HD22: 4A: 1: O: | 2.092 |  |
|  |  |  |  |  |  |  |  |  |  |  |  | 4csd:A : ASN 138: HD22: 4A: 1: O: | 2.231 |  |
|  |  |  |  | | |  |  |  |  |  |  |  |  |  |
|  |  |  |  | | |  |  |  |  |  |  | 5C: 1:H: 4csd: A: SER 148:O: | 1.886 |  |
| 4c |  | | -7.385 | | | -0.29 | 13.81 | -7.13 | 2 |  |  | 4csd:A : ASN 138: HD22: 4A: 1: O: | 2.113 |  |
|  |  |  |  |  |  |  |  |  |  |  |  |  |  |  |
|  |  | | |  |  |  |  |  |  |  |  |  |  |  |
|  |  | | |  |  |  |  |  |  |  |  | 4csd:A : ALA 189: HN: 5D: 1: S: | 1.841 |  |
| 4d |  | | |  | -7.812 | -0.37 | 2.34 | -8.33 | 3 |  |  | 5D: 1:H: 4csd:A : ALA 189:O | 2.071 |  |
|  |  | | |  |  |  |  |  |  |  |  | 4csd:A : THR 190: HG1: 5D: 1: N | 2.098 |  |
|  |  | | |  |  |  |  |  |  |  |  | 4csd:A : ALA 40: HN: 5E: 1: O: | 1.726 |  |
| 4e |  | | |  | -7.319 | -0.35 | 1.33 | -861 | 3 |  |  | 4csd:A : TRP 81:HE1 5E: 1: O: | 1.922 |  |
|  |  | | |  |  |  |  |  |  |  |  | 5E: 1: H: 4csd:A : GLU28: OE1 | 2.126 |  |
| 4f |  | | |  |  |  |  |  |  |  |  | 5H: 1: H: 4csd:A : ALA 7: O: | 2.037 |  |
|  |  | | |  | -7.966 | -0.35 | 3.66 | -8.19 | 3 |  |  | 4csd:A : THR 51:HG1 5H: 1: N: | 2.054 |  |
|  |  | |  | | | | | | |  |  | 5G: 1:H: 4csd: A: ALA 98: O:  4csd:A : THR 51: HG1: 5G: 1: N | 2.038  2.194 |  |
| 4g |  | | -7.823 | | | -0.19 | 45.13 | -8.61 | 2 |  |  |  |  |  |
|  |  |  |  |  |  |  |  |  |  |  |  |  |  |  |
|  |  | | |  |  |  |  |  |  |  |  |  |  |  |
| 4h |  | | -6.970 | | | -0.33 | 11.54 | -8.15 | 1 |  |  | 4csd:A : THR 51: HG1: 5F: 1: O: | 2.154 |  |
|  |  |  |  |  |  |  |  |  |  |  |  |  |  |  |
|  |  | | |  |  |  |  |  |  |  |  |  |  |  |
| 4i |  | | |  | -7.744 | -0.34 | 3.23 | -8.09 | 2 |  |  | 5I: 1:H: 4csd: A: ALA 98: O: | 1.980 |  |
|  |  | | |  |  |  |  |  |  |  |  | 4csd:A : ALA 98: HN: 5I: 1: S: | 2.106 |  |
|  |  | | |  |  |  |  |  |  |  |  |  |  |  |
|  |  | | |  |  |  |  |  |  |  |  | STREPTOMICIN: 1: H: 4csd:A :ALA 98:O: | 2.058 |  |
|  |  | | |  |  |  |  |  |  |  |  | 4csd:A :GLY102:HN: STREPTOMICIN: 1:O: | 2.061 |  |
| Standard |  | | |  | -6.466 | -0.09 | 2.90 | -8.24 | 5 |  |  | STREPTOMICIN: 1:H : 4csd:A: VAL 141:O: | 2.067 |  |
|  |  | | |  |  |  |  |  |  |  |  | STREPTOMICIN: 1:H: 4csd:A : ALA 7:O: | 2.168 |  |
|  |  | | |  |  |  |  |  |  |  |  | STREPTOMICIN: 1:H : 4csd:A: VAL 141:O: |  |  |
